# Two disjunct Pleistocene populations and anisotropic postglacial expansion shaped the current genetic structure of the relict plant *Amborella trichopoda*

**DOI:** 10.1371/journal.pone.0183412

**Published:** 2017-08-18

**Authors:** Rémi Tournebize, Stéphanie Manel, Yves Vigouroux, François Munoz, Alexandre de Kochko, Valérie Poncet

**Affiliations:** 1 UMR DIADE, Institut de Recherche pour le développement, University of Montpellier, Montpellier, France; 2 UMR CEFE, Ecole Pratique des Hautes Etudes, PSL Research University, CNRS, University of Montpellier, Montpellier SupAgro, IRD, INRA, Montpellier, France; 3 UMR AMAP, University of Montpellier, Montpellier, France; 4 French Institute of Pondicherry, Pondicherry, India; National Cheng Kung University, TAIWAN

## Abstract

Past climate fluctuations shaped the population dynamics of organisms in space and time, and have impacted their present intra-specific genetic structure. Demo-genetic modelling allows inferring the way past demographic and migration dynamics have determined this structure. *Amborella trichopoda* is an emblematic relict plant endemic to New Caledonia, widely distributed in the understory of non-ultramafic rainforests. We assessed the influence of the last glacial climates on the demographic history and the paleo-distribution of 12 *Amborella* populations covering the whole current distribution. We performed coalescent genetic modelling of these dynamics, based on both whole-genome resequencing and microsatellite genotyping data. We found that the two main genetic groups of *Amborella* were shaped by the divergence of two ancestral populations during the last glacial maximum. From 12,800 years BP, the South ancestral population has expanded 6.3-fold while the size of the North population has remained stable. Recent asymmetric gene flow between the groups further contributed to the phylogeographical pattern. Spatially explicit coalescent modelling allowed us to estimate the location of ancestral populations with good accuracy (< 22 km) and provided indications regarding the mid-elevation pathways that facilitated post-glacial expansion.

## Introduction

Climatic fluctuations have prevailed throughout the Quaternary period, influencing species distribution and population dynamics through an alternation of expansion, contraction and fragmentation phases [1, 2]. These dynamics have shaped the current distribution and structuring of intra-specific genetic diversity [3]. Specifically, isolation of small populations within refugial habitats during past glacial periods could increase genetic drift and favour population divergence [4], both influencing the present-day pattern of genetic diversity. Modelling species’ demographic history from present patterns of genetic diversity should then allow identifying the influence of these past dynamics [5, 6]. Based on coalescent theory, demo-genetic models allow inferring the plausible past demographic and migration dynamics that shaped the distribution of neutral genetic polymorphisms observed in current populations [7]. In particular, the inferred variations in effective population size *N*_*e*_ can reveal the imprint of fluctuating climatic conditions on species’ demographic and range dynamics [8].

**Fig 1:**
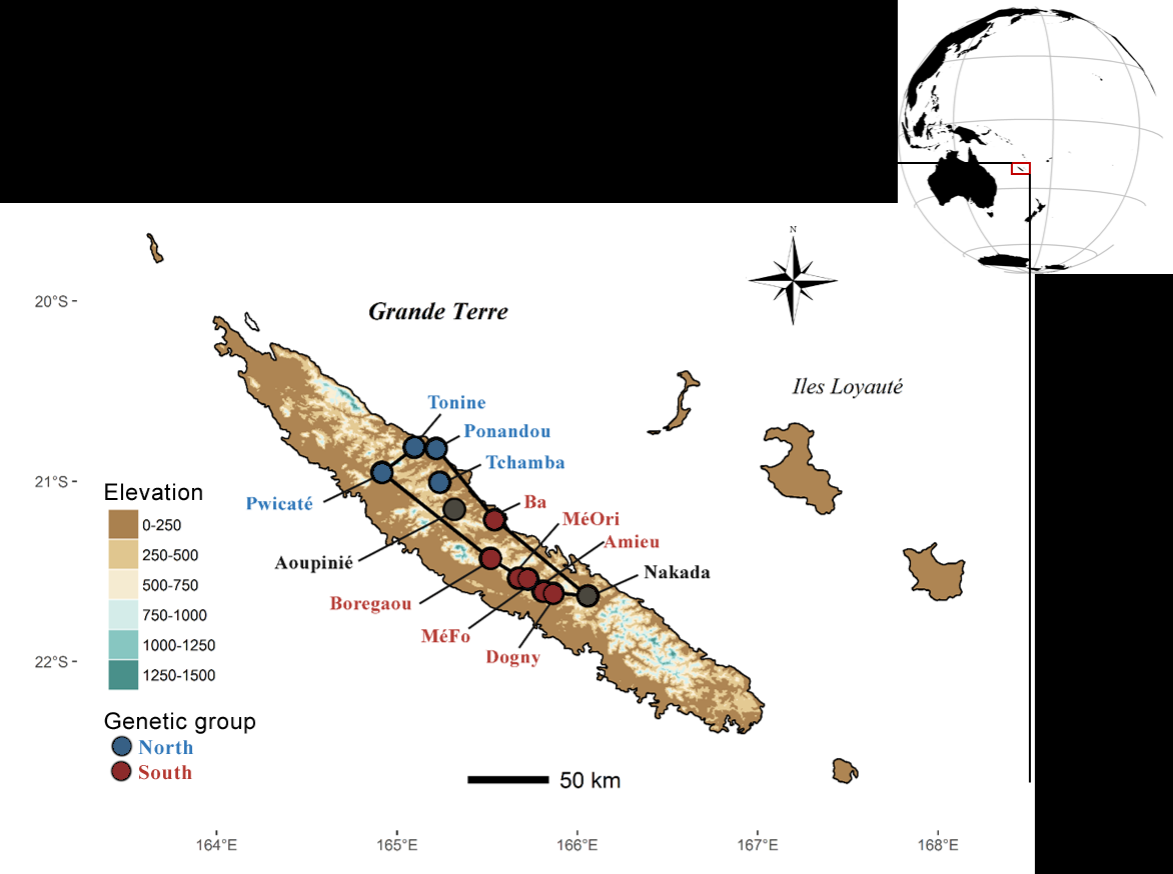
Topography of New Caledonia and geographical distribution of the 12 studied populations spanning the whole natural distribution of *Amborella trichopoda* at present (black line polygon) [13]. Black points represent populations present in both re-sequencing and microsatellite datasets; blue points in the re-sequencing dataset only; red points in the microsatellite dataset only. Population names are coloured according to their genetic group assignment (blue for the North group, red for the South, black when undetermined). The topography of New Caledonia ranges from sea level up to 1,630 m ASL (Mont Panié). *Amborella* currently grows between 100 and 1,000 m ASL.

We designed a demo-genetic approach to assess the past demographic history and the range dynamics of *Amborella trichopoda* Baill., a flowering plant endemic to New Caledonia. New Caledonia is an archipelago located in the southwestern Pacific Ocean (Fig 1) and a biodiversity hotspot where more than 74% of the 3,099 inventoried angiosperm species are endemic [9]. High environmental heterogeneity prevails in this country [10] and the climate—currently tropical to sub-tropical—has greatly fluctuated over the last 30,000 years [11, 12]. Some studies have suggested the presence of paleo-refugia for rainforest species during the LGM period [13–15]. However, very few studies have been carried out on the population genetics of New Caledonian plants [16–20] and only one concerned a rainforest species [13]. *Amborella trichopoda* is an emblematic understory shrub and a relevant model species to assess the biogeography of New Caledonian rainforests [15]. Furthermore, *Amborella* is the most basal clade of angiosperms, and previous studies have investigated its genome and genetic diversity in order to understand the evolution of flowering plants [21–25]. Recent microsatellite marker studies [13, 26] revealed well-differentiated and geographically distinct genetic groups. Moreover, the present geographical distribution of *Amborella* in New Caledonia suggests that climate change had an impact during the LGM (ca. 22,000 years BP) and Holocene (ca. 12,000 years BP), when *Amborella* experienced a dramatic reduction in suitable habitat. The survival and expansion of at least two lineages from putative refugia might have generated the major diversity groups [13].

In this study, we aimed to spatiotemporally hindcast the past demography of *Amborella* populations, by: (1) assessing the number and age of ancestral populations which contributed to its current genetic structure, (2) geographically mapping these ancestral populations, and (3) characterizing the post-glacial population range expansion, while estimating the dispersal capacities and habitat suitability.

We thus combined non-spatial demographic inference and spatiotemporal simulation of population range expansion in a heterogeneous environment. We characterized post-glacial population expansion from the locations of the ancestral populations. We compared and discussed the inferred spatial distribution of ancestral populations and the population dynamics with the *Amborella* potential habitat maps estimated using Species Distribution Modelling (SDM). While the theoretical and methodological foundations of spatially explicit demo-genetic modelling have previously been investigated [27–29], to our knowledge this is the first study proposing an original and comprehensive modelling approach to map ancestral populations and subsequent expansion dynamics in a tropical environment.

## Material and methods

### Study system

The study area is located on the main island (*Grande Terre*) of New Caledonia (Fig 1) in the southwest Pacific Ocean. *Amborella trichopoda* belongs to a monotypic genus endemic to the island and distributed throughout most of the central part in the understory of non-ultramafic rainforests. The species is a dioecious shrub probably pollinated by wind or insects [30]. The current natural distribution of *Amborella* (Fig 1) has been precisely documented over the last 20 years [13, 15, 30–33], and we surveyed populations sampled throughout this distribution.

### Genetic data

We used two genetic datasets: a whole-genome resequencing dataset consisting of 12 individuals sampled among 11 populations (see S1 Table) for genetic structure analysis and non-spatial demographic inferences, and a microsatellite dataset consisting of 11 populations (222 individuals) genotyped on 10 markers from Poncet et al. [13] for spatial demographic inferences.

The whole-genome resequencing dataset was used to analyse genetic admixtures and to infer the existence and number of ancestral populations. Raw sequences were mapped onto the reference genome of *A*. *trichopoda* [32] using a bioinformatic pipeline described in S1 Appendix.

The potential spatial distribution of the ancestral populations was then analysed using the microsatellite dataset with alternative spatially-explicit modelling. This dataset had a larger sampling size.

### Genetic structure analysis

To determine the genetic structure, we applied an unsupervised clustering algorithm, sNMF [34] to a filtered set of 100k single nucleotide polymorphisms (SNPs) derived from the whole-genome resequencing dataset (S1 Appendix). The most likely number of genetic clusters (*K*) was determined as the one minimizing the cross-entropy criterion, for *K* ranging between 1 and 10 (with 20 estimation runs per *K*). Cluster number and individual admixture coefficients were confirmed using the clustering approach implemented in ADMIXTURE [35] (S1 Fig) and compared to the genotype likelihood-based approach implemented in NGSAdmix [36] (S2 Appendix). Weir & Cockerham’s pairwise *F*_*ST*_ was computed and tested for significance by performing 999 permutations of individuals (S2 Appendix).

Based on the most probable number of genetic clusters (*K* = 2), whole-genomic SNPs were summarized in a joint probabilistic two-dimensional and folded site frequency spectrum (2D-SFS) computed by taking genotyping uncertainty into account with the ANGSD 0.9 software package [37]. This probabilistic approach was preferred to limit the bias that low-coverage sequencing entails on the calls of heterozygous sites. Only high-confidence sites in intergenic regions were considered and genotype likelihoods were estimated assuming the SOAPsnp sequencing error model, resulting in a probabilistic 2D-SFS containing ~120k SNPs (Li et al. 2009b) (S1 Appendix). The SFS was represented as a two-dimensional matrix where any cell of *xy*-coordinates quantified the number of SNPs found at minor frequencies *x* and *y* in the first and second population, respectively.

### Inferring the demographic history and the number of ancestral populations

To determine the species demographic history and the past number of ancestral populations, we compared the maximum composite-likelihood of 2D-SFS among alternative, coalescent-based demo-genomic models, using *fastsimcoal* 2.5.2.11 [38, 39]. Alternative models considered the way climatic fluctuations of the Quaternary period could have influenced the species demography through alternation of population contraction and expansion from ancestral populations. We postulated that the last contraction event was characterized by a reduction in population sizes compared to present, followed by population expansion. We thus compared twelve demographic scenarios—assuming either a single or two past ancestral population(s) present during the potential contraction phase, out of which the two currently major genetic groups could be derived (S2 Appendix). In addition to the number and size of putative ancestral populations, the twelve demographic scenarios also considered different divergence time between the two major genetic groups. We also considered variations in expansion dynamics: (a) abrupt or smooth population growth, and (b) presence or absence of gene flow among populations before and/or during the expansion phase (S3 Table). Parameters of each model are described in S2 Appendix.

The most likely model was selected after calculating the maximum composite likelihood of all models. For each model, composite likelihoods were maximized in 120 independent runs using random initial parametric seeds. Model fits were compared based on the best likelihood estimate for each model based on the Akaike Information Criterion (AIC) evidence ratio.

For the selected model, confidence intervals for each estimated parameter were computed using parametric bootstraps based on 100 predicted SFSs [39]. The predictive power of the model was assessed by generating DNA polymorphisms along 10^5^ independent sequences of 300 bp each, corresponding to the total length of sequences analysed for the joint spectrum construction using the maximum likelihood point estimates. Observed and predicted polymorphisms were then compared using a set of within/between population genetic summary statistics (S2 Appendix).

The maximum number of ancestral populations tested in the models was limited to two populations because of lack of power to identify models with higher number of ancestral populations (S2 Appendix). Moreover, to account for the possible impact of current unsampled populations onto the demographic inference, a supplementary structured model was designed, including three additional unsampled populations to the two previously sampled ones. In this model, the five current populations diverged at the same time and the sampled populations were allowed to exchange symmetric gene flow with the unsampled ones after the expansion onset (S2 Appendix).

### Localizing ancestral populations and characterizing expansion dynamics

To infer the geographical extent of the ancestral populations and their recent expansion, we carried out spatially explicit, coalescent demo-genetic modelling using the microsatellite marker dataset. This modelling complements the previous demo-genetic model by taking into account the spatiotemporal range of expansion and the isolation-by-distance. In addition, we acknowledged landscape heterogeneity in spatially-explicit dynamics. Using SPLATCHE 2.01 [28], we (1) inferred the spatial coordinates of ancestral populations before expansion, and (2) characterized subsequent population range expansion dynamics.

We simulated dynamics based on two randomly sampled locations of ancestral populations (henceforth referred to as “origin locations”) and a set of: (a) ecological parameters (topography-dependent resistance to gene flow, habitat carrying capacities), (b) demographic parameters (intrinsic migration and logistic growth rates of populations), and (c) genetic parameters (mutation rate and gamma distribution of mutational transitions between microsatellite repeat counts) (S2 Appendix). To limit model complexity, the values of two parameters were fixed according to the estimates derived from the previous non-spatial demographic model: the starting age of expansion (12,800 years BP), and the age of divergence between the Northern and Southern groups (23,400 years BP). Additional cross-validation studies were performed to test the robustness of the origin location estimates to these timing estimates (S2 Appendix).

First, the expansion of the ancestral populations was simulated across the landscape forward in time, considering that each focal pixel of the landscape could include a local population growing logistically with intrinsic growth rate *r* and carrying capacity *K*_*i*_, defined as the maximal number of individuals that the pixel could sustain. As a trade-off between computation load and the quality of local topographic feature representation, the pixel size was set to 1.73 km². Each population could send *m∙N*_*e*_(*t*) migrants to the four cardinally adjacent pixels at each generation. This model corresponds to a 2D stepping-stone model, with *m* being the intrinsic stepwise migration rate, and *N*_*e*_(*t*) being the size of the local population at any time *t* after the expansion onset. The proportion of migrants sent to each neighbouring local pixel was determined by the mean elevation in these adjacent pixels, so migration could be influenced by the topographical context (S2 Appendix). Carrying capacities were partitioned into three latitudinal areas subdividing the island, referred to as the “Northern”, “Central” and “Southern” zones. The three pre-defined zones were demarcated *a priori* along two deep valleys crossing the central mountain chain (Ponérihouen and Houaïlou). From North to South, they included 2,283; 532 and 1,475 pixels and partitioned the current species distribution in almost equal proportions (35%, 28% and 36% of the distribution, respectively).

Carrying capacities could vary across these zones but were considered temporally constant within. Preliminary model comparisons suggested that using independent carrying capacities for each of these three pre-defined zones improved the fit of the spatial model.

Once the expansion dynamics reached present time, individuals simulated at present time were sampled at the same geographic coordinates as the observed samples, and a stochastic coalescent graph was tracked backward in time for these individuals. Using a generalized stepwise model of genetic mutation, we thus simulated microsatellite polymorphisms conditionally to the population range expansion history.

Parameters were estimated using Approximate Bayesian Computation (R package *abc*, Csilléry et al. [40]): we simulated 600,000 microsatellite datasets based on independent draws from parametric prior distributions in order to estimate their posterior marginal distributions. Polymorphisms were summarized by a set of 159 genetic statistics, including within-population diversity indices and between-population differentiation indices (S2 Appendix). The dimensionality of summary statistics was reduced using neural networks. Posterior parameter distributions were inferred based on a 0.5% tolerance ratio.

Parameter estimation accuracy was assessed using a total of 100 local leave-one-out cross-validations (S2 Appendix). Estimation performance was assessed using the great-circle distance between true and estimated locations of origins and the mean-Standardized Root-Mean-Square Error (SRMSE) for all parameters. To check whether the posterior model actually predicted microsatellite polymorphisms fitting the observed ones, we performed a predictive model check by simulating 2,850 datasets from the posterior parameter distribution. A goodness-of-fit test was then carried out under the null hypothesis that observed summary statistics fell within the range of simulated statistics.

We further checked the robustness of the estimates for the ancestral population locations to the inclusion/exclusion of additional ancestral populations and to distinct patterns of carrying capacities (one *vs*. three pre-defined zones). Details are given in S2 Appendix.

### Past species distribution modelling

Habitat suitability at the location of putative past populations were modelled under the mid-Holocene (*~*6,000 years BP) and the Last Glacial Maximum (LGM, *~*18,000 years BP) by projecting the current distribution model of *A*. *trichopoda* as determined by Poncet et al. [13], using the MaxEnt algorithm [41]. LGM and mid-Holocene were two periods of major climate-driven vegetation changes in New Caledonia [42].

PMIP2 paleoclimatic layers were downloaded with a resolution of 2.5 arc-minutes from the WorldClim platform [43], considering four different general circulation models (GCMs): CCSM4, MIROC-ESM, MPI-ESM and IPSL-CM5A. For paleo-niche projection, we used a set of 17 environmental predictors: annual precipitation, all BIOCLIM temperature variables and a set of variables assumed to be temporally static (soil type, digital elevation, slope, eastness and northness indices) (S2 Appendix).

## Results

### North-South disjunction in genetic structure

Genetic structure analyses based on whole-genome resequencing dataset (100k SNPs) supported a phylogeographic pattern consisting in two main (K = 2) latitudinally distinct groups, henceforth denoted as “North” and “South” groups (S1 Fig). These two groups are well differentiated (Weir & Cockerham’s *F*_*ST*_ = 0.46; *P* < 0.002). The resequenced Aoupinié individual located at 21.08° latitude was excluded from the non-spatial genomic analysis due to a North group assignment inconsistent with previously published structure [13] that might have biased the inference. However, the whole Aoupinié population (27 individuals in the microsatellite dataset) was included in the spatial analysis which did not require genetic group assignment.

### Number, age and demography of ancestral populations

**Fig 2:**
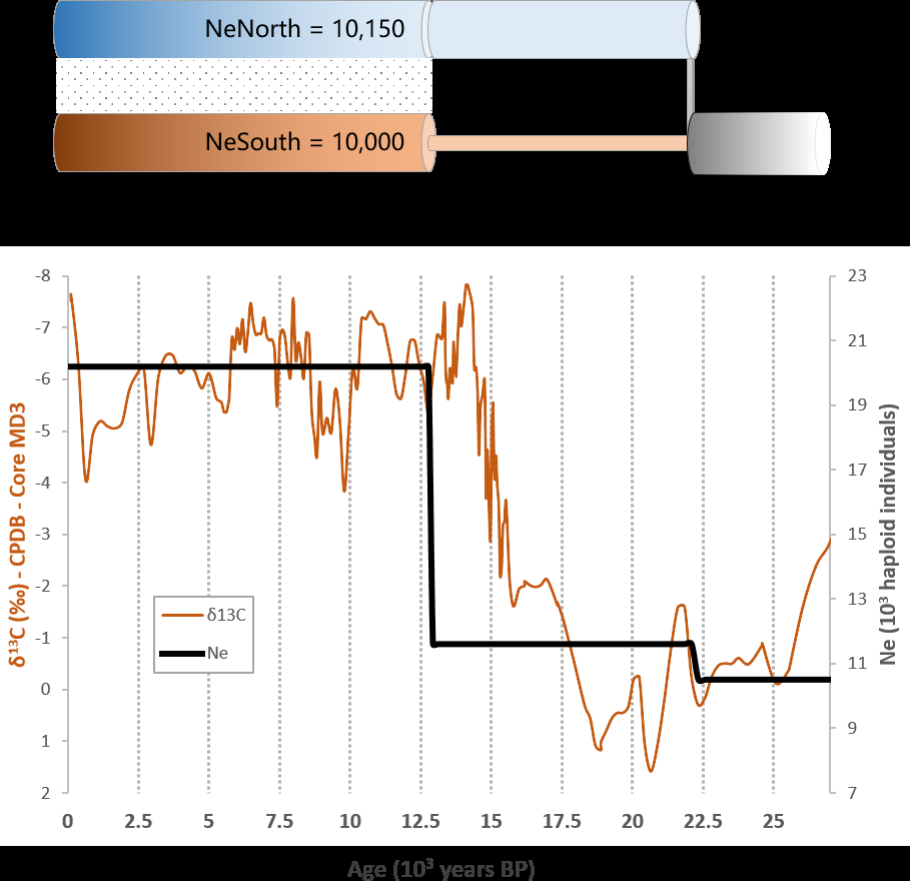
Variations in *Amborella trichopoda* effective size and variations in the δ^13^C isotopic ratio. (**A**) **Best demo-genomic model (*2M*)** estimated from the whole-genomic sequence dataset. The radii of demographic pipes are scaled by the corresponding effective population sizes. The dotted interspace depicts gene flow. (**B**) **Variations in the effective size of *Amborella*** (black solid line) from present time to 25,000 years BP as estimated from the best demo-genomic model. The orange solid line (left *y*-axis) is the smoothing spline of the δ^13^C isotopic ratio obtained from Hellstrom et al. [44], a proxy of dense lowland forest productivity in Northern New Zealand, ~2,000 km south of New Caledonia. Consistent demographic and δ^13^C isotope ratio variation trends suggest a positive lag time between the post-refugial expansion of *Amborella* and the re-expansion of forests in Northern New Zealand.

The most likely demographic scenario (model *2M*, Fig 2) suggested that an earliest population of *Amborella* split into two isolated ancestral populations during the late glacial period followed by a species-wide size increase with gene flow. This model was supported by a strong evidence ratio relative to the 11 other tested demographic models (S2 Table). It accurately predicted observed polymorphism: exact prediction of the observed *F*_*ST*_, and of the fraction of singletons in North (38%) and South (33%) populations (S6 Table).

Using an estimated generation time of four years (Amborella Genome Project 2013), we estimated that the two ancestral populations diverged 23,400 [13,700–87,770] years BP and remained isolated with constant size until 12,800 years BP [6,500–39,40]. Demographic expansion occurred after 12,800 years BP with a 6.3-fold expansion of the Southern population, which grew from an effective size of 1,550 [51-9,675] haploid individuals to 10,000 [3,680–38,560], while the size of the Northern population was relatively stable, *i*.*e*. from 10,100 [2,172-29,985] to 10,150 [4,50-38,40] individuals. From 12,800 years BP onwards, weak and asymmetric gene flow was estimated between the groups. The Northern population sent around 3-fold more migrants to the South than the reciprocal.

We compared the species-wide demographic variations of *Amborella* with the variations of the δ^13^C isotopic ratio measured in a record of cave mineral deposits from New Zealand (Hellstrom et al. [44]; Mt. Arthur, ~2,000 km south of New Caledonia), which were negatively correlated with productivity changes in dense lowland forest (Fig 2). We found that *Amborella* re-expanded in New Caledonia about two millennia after rapid forest re-expansion in northern New Zealand, ~14,000 years BP.

### Location of ancestral populations and expansion dynamics

**Fig 3:**
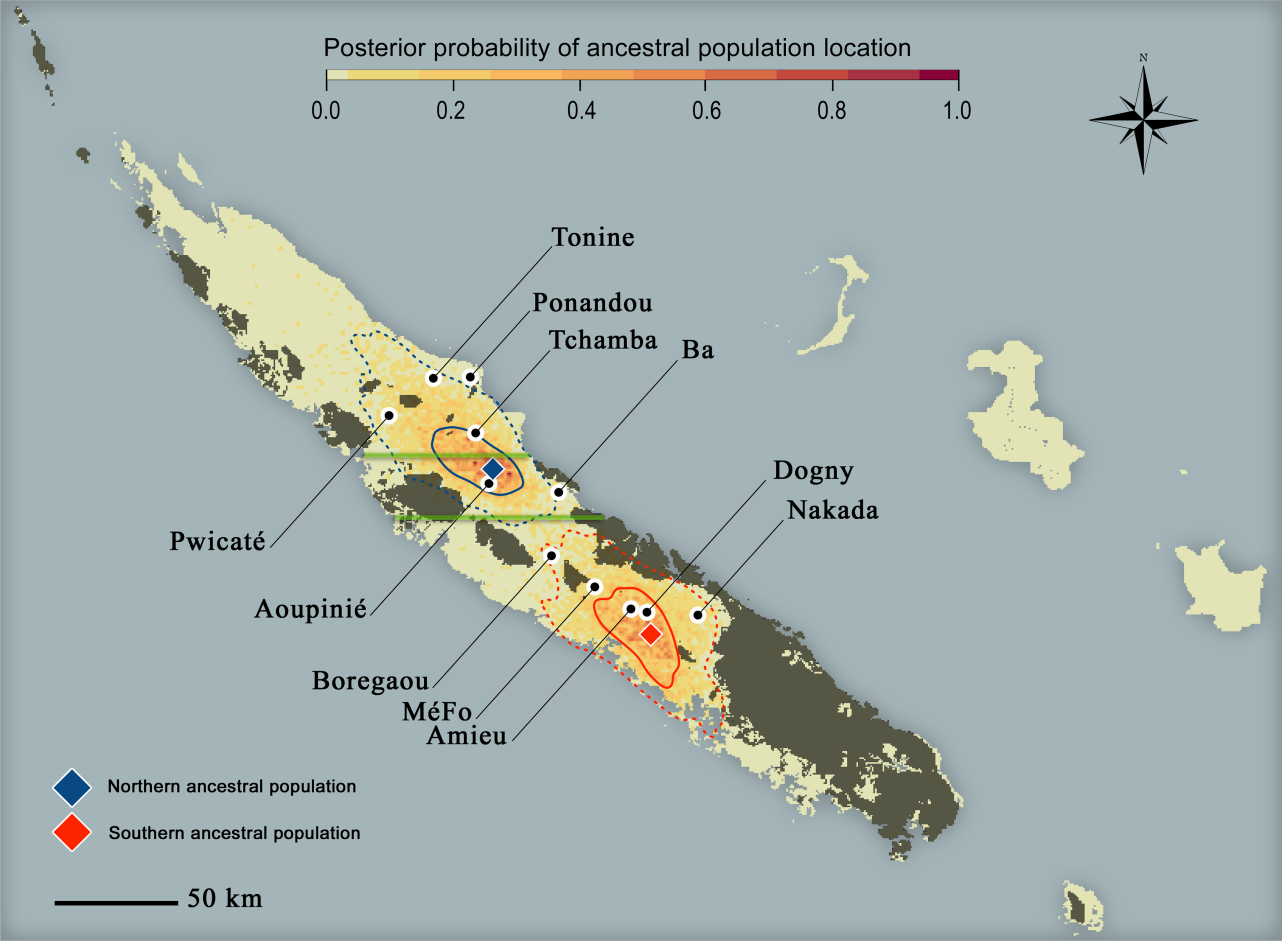
Putative *Amborella* ancestral population locations at 12,800 years BP, as inferred from the spatially explicit modelling. Solid curves define the envelope containing 50% of the posterior location density for each ancestral population, while dotted curves define the 95% envelopes. Diamonds indicate the location with maximum posterior probability for each refugium: 21.70°S–165.88°E for the South, 12 km southwards to Dogny; 21.13°S–165.30°E for the North, 5 km northwards to Aoupinié. The two 50% envelopes illustrate that ancestral populations were likely spread over narrow areas: the Northern population likely occupied the Aoupinié Mount and areas southwestward to Tchamba valley; while the Southern population occupied Dogny Plateau, Rembai Mount, Amieu and some hills eastwards from La Foa. The two horizontal green lines delimit the “Northern” (*K*_*N*_), “Central” (*K*_*C*_) and “Southern” (*K*_*S*_) zones of habitat carrying capacity. Darker areas correspond to ultramafic substrates where *Amborella* does not grow. Black dots indicate the location of the 11 sampled sites.

Spatial demographic inference suggested that the populations of *Amborella* were split into two spatially distinct areas before the expansion phase (starting at ~12,800 years BP) (Fig 3): a northern origin located in the Aoupinié region, and a southern one in the vicinity of the Dogny Plateau. Maximum density locations for these origins of expansion were separated by a distance of 90 km, and the 95% credibility envelope did not intersect, suggesting clear genetic and geographical disjunction of ancestral populations. Based on local leave-one-out cross-validations, we estimated the ancestral population location in a median great-circle-distance of 21.8 km for the North and 17.0 km for the South. Moreover, the estimation of the expansion origin coordinates appeared robust to the variation of the time parameters (age of divergence and expansion onset) and to the properties of the carrying capacity map. We observed no significant bias when including or excluding origins of expansion (S2 Appendix).

During the expansion phase (12,800–0 years BP), carrying capacities in both Northern and Central non-ultramafic zones have been around 20-fold higher than in the Southern region (S8 Table). Comparisons of carrying capacities between Northern and Central parts were challenging due to the large credible intervals (S2 Fig). Colonized local populations started growing with a ~5-fold [0.5–9.6] proportional increase of the effective size *N*_*e*_ in one generation, before slowing down and reaching a plateau at the specific carrying capacities (S8 Table). The effective migrations rates in the Northern, Central and Southern zones were 1.37, 2.03 and 0.06-fold the area-averaged rate, respectively. Concerning the influence of topography on migration, gene flow was restricted above ~600 m elevation, and there was 36-fold less chance that an allele could flow 100 m downward than it could flow 100 m upward (S8 Table). The estimated elevational threshold was close to the mean elevation of the current distribution, *i*.*e*. 594 ± 96 m [13]. Some expansion-related parameters could be accurately estimated (SRMSE < 25%) and included the expansion origin coordinates, the migration rate and the Southern carrying capacity. The SRMSE of other expansion-related parameters ranged between 57 and 83% (S7 Table). Low accuracy concerned parameters related to local population growth and topography resistance to gene flow.

The posterior adjusted model correctly predicted observed microsatellite summary statistics with more than 96% of the 159 observed summary statistics falling within the range of values predicted by the model.

### Paleo-habitat models

The potential habitat of *Amborella* under the Last Glacial Maximum (LGM, ~21,000 years BP for PMIP2) varied noticeably depending on the general circulation model (GCM) we used (S4 Fig). Compared to present-day, the suitable area (i.e. suitability higher than the equal training sensitivity and specificity threshold) was reduced by 7% under MIROC, and more than 98% under CCSM or MPI. MIROC predicted slightly wetter climate than other GCMs, with stronger temperature seasonality than present. The pattern of expansion origins was compatible with the map of habitat suitability at ~21,000 years BP inferred using the MIROC GCM, considering the stability of the ancestral population locations during the 23,400–12,800 years BP contraction phase.

**Fig 4:**
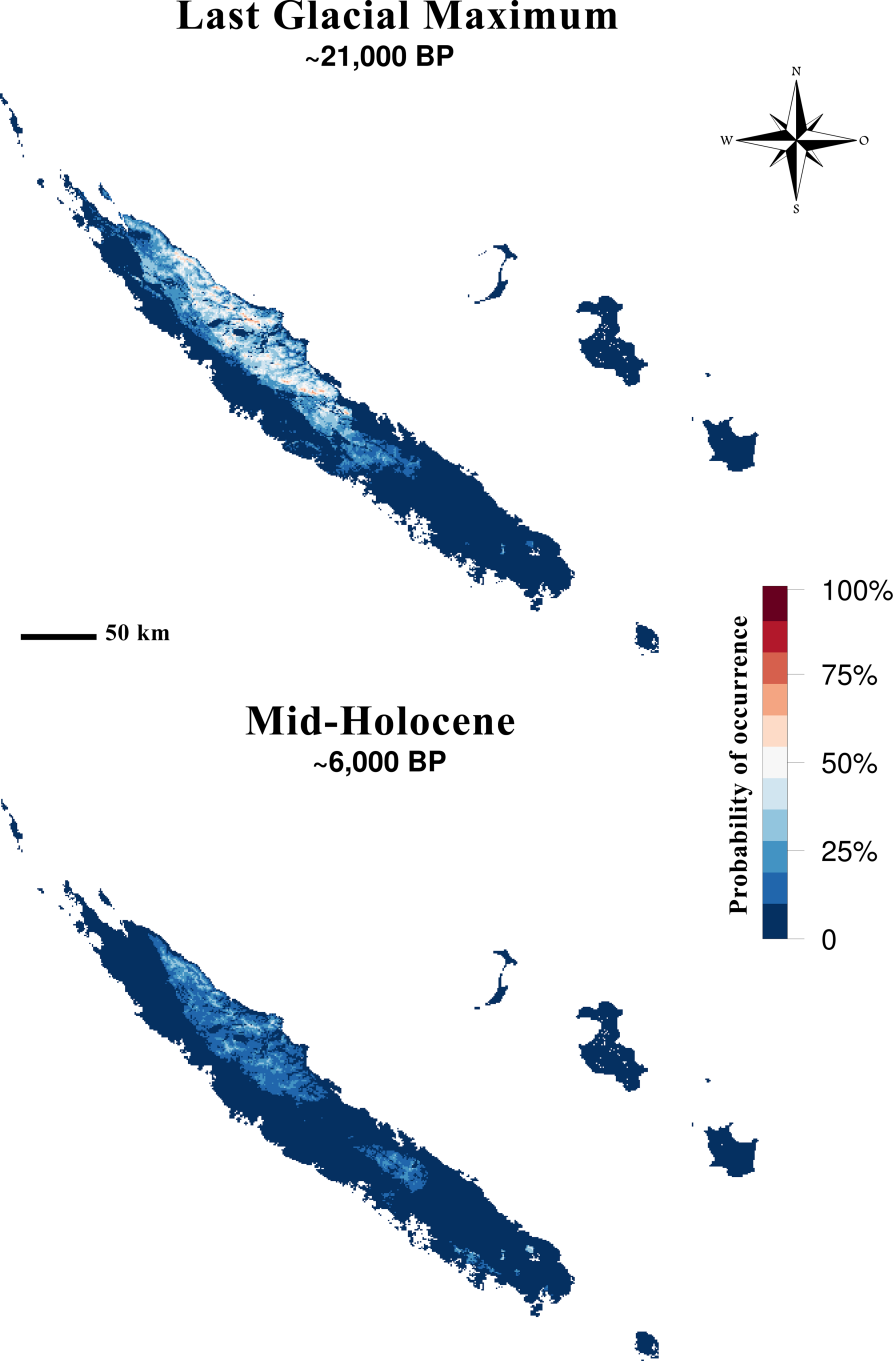
**Habitat suitability for *Amborella* in New Caledonia** under (**A) the Last Glacial Maximum**, ~21,000 years BP and (**B) the mid-Holocene**, ~6,000 years BP. Paleo-habitat projections were performed using MIROC paleo-climatic layers.

At mid-Holocene, MIROC-based niche projection predicted a 98% reduction in suitable area compared to present, which was therefore even more severe than during the LGM. This reduction affected particularly the Southern zone (Fig 4). For CCSM and MPI GCMs, climate conditions at mid-Holocene were out of the training ranges on a large part of *Grande Terre* (S4 Fig), thus limiting the interpretation of corresponding suitability predictions. But whatever the GCM used, paleo-habitat projections predicted severe habitat constriction for *Amborella* during the mid-Holocene compared to present day [13].

## Discussion

We found that an early population of *Amborella* could have split into two distinct Pleistocene ancestral populations about 23,400 years BP, one in the North and another in the South. The latter mapped to an area never detected in previous studies as a refugium candidate for non-ultramafic rainforest plant species. The South ancestral population expanded 6.7 fold while the size of the North population remained stable until 12,000 years BP. Then an asymmetric gene flow from North to South was observed leading to the current genetic structure in two groups with admixture. This pattern of expansion origin was compatible with the map of habitat suitability derived from past distribution models.

### Likely scenarios and inference of divergence and expansion timing

In the demographic model, we could reliably test only the assumption of one or two ancestral populations. A scenario assuming more than two ancestral populations for the Northern contemporary population was tested but could not be statistically distinguished from models assuming exactly two ancestral populations (S2 Appendix). The alternative models designed to approximate a subdivision during the expansion phase (23,400–12,000 years BP) by taking into account gene flow with putatively unsampled populations did not significantly change the previous estimates of expansion onset age and of the different expansion intensity between the Northern and the Southern populations (S2 Appendix).

Demographic model inference suggested that the ancestral populations diverged between 13,700 and 87,800 years BP (95% confidence interval) and that the Southern population started expanding between 6,500 and 39,000 years BP. The confidence intervals of divergence and expansion timings took into account the uncertainty of the mutation rate. The age of first reproduction was a proxy of the generation time, probably underestimating actual generation time [45] which could be twice as large, displacing the lower bound of the expansion onset to the last stage of the glacial period. Likewise, seed dormancy [46] might affect the estimations. For *Amborella*, no fossil data and no reliable estimates of neutral substitution rate are currently available. Owing to the uncertainty of mutation rate estimates, the parameter was left as a free parameter in our modelling, bounded between 5·10^−9^ and 1·10^−7^ substitutions·b^-1^·g^-1^. Our point estimate was higher (4.15·10^−8^) than the estimate based on mutation-accumulation lines of the eudicot *Arabidopsis thaliana* (7·10^−9^; [47] but closer to the *Amborella* estimate of substitution rates (1.41·10^−8^) proposed by the Amborella Genome Project [32].

### Demographic dynamics and paleo-climatic variations

The size of the early population was low *prior* to divergence, about half of today’s cumulated population size, with a pre/post-divergence size ratio of 11/10. Based on our temporal point estimates, this past restriction was contemporary with significantly drier climate in New Caledonia around 26,000 years BP [11]. The two daughter ancestral populations resulting from the divergence could have been constrained spatially by lesser availability of suitable habitat. The 23,400–12,800 years BP period coincided with the late glacial period in New Caledonia, stretching over the Last Glacial Maximum (LGM, ~21,000–18,000 years BP) until the Pleistocene-Holocene transition (~14,000–9,000 years BP) [42].

The population size of the Northern group after divergence remained constant, with an estimated size of around 96% of the ancestral population, suggesting temporal stability of the Northern ancestral population size *ante* 23,400 years BP. Abundance stasis was previously expected for organisms from ocean islands, owing to the buffering role of the surrounding ocean [48]. On the contrary, the Southern ancestral population experienced strict genetic bottleneck (compared to its present size) and would suggest a context of classical refugium [48].

Our estimations of the *Amborella* cumulated size contraction during the 23,400–12,800 years BP period were in line with palynological evidence of rainforest contraction in the Southwest Pacific region at the Pleistocene-Holocene transition [12]. *Amborella* is associated with the understory of non-ultramafic rainforests and oscillation of rainforest cover sets an upper boundary on the specific distribution of *Amborella*. Other studies based on isotopic records from New Zealand also suggested major vegetation changes in the region during the LGM compared to present (Fig 2) [44]. In New Caledonia, Southern lowland forests were enriched with montane taxa about 20,000 years BP, supporting the existence of a glacial period with temperature and rainfall depression [42, 49]. Furthermore, the glacial to interglacial transition has been recognised as being environmentally instable, with dramatic vegetation changes associated with fire events, in particular about 14,000 years BP, a few centuries before our estimated age of re-expansion for *Amborella* [42]. Major climate disturbance associated with the Pleistocene-Holocene transition could have fostered the genetic differentiation of *Amborella* by driving the fragmentation of its distribution into two separate ancestral populations from 23,400 to 12,800 years BP.

### Geographical locations of the ancestral populations

Spatially explicit modelling using microsatellite markers allows pinpointing the geographical extent of population expansion origins, accounting for landscape heterogeneity and the effect of unsampled local populations. Our spatial model displayed two separate geographical origins between ~23,400 and ~12,800 years BP (Fig 3). Post-hoc tests suggested that the estimated locations were not biased by the a priori number of ancestral populations implemented in the spatial model (S2 Appendix).

Their locations were predicted in suitable habitats under the MIROC SDM, while the area between the two geographic regions was predicted to have been unsuitable at the mid-Holocene. The two ancestral populations then stood on both sides of the Houaïlou River valley, likely an unsuitable area for *Amborella* about 12,800 years BP. Unsuitable microclimatic conditions in this area during the LGM and the glacial-interglacial transition could have created and maintained an ecological barrier disrupting gene flow between the Northern and Southern groups and promoting their divergence. Owing to the time estimate uncertainty for the expansion onset (6,500–39,040 years BP), the distribution of the ancestral population locations could be also compatible with the pattern of suitable habitat at mid-Holocene (~6,000 years BP).

The Northern ancestral population origin was included within an area that was already identified as a refugium for sixty relict angiosperm taxa predominantly restricted to non-ultramafic rainforests under the LGM [15]. Based on CCSM paleo-projections of their species distribution models, Pouteau *et al*. (2015) found maximum species richness under LGM in the Aoupinié Mount area, which is in line with our predicted location of maximum presence probability for the Northern ancestral population of *Amborella*. Although the Massif des Lèvres has been suggested as a putative refugium in a preliminary study [13], our modelling allowed to pinpoint more accurately the most likely origin within the probability envelopes.

Moreover, our spatial demo-genetic modelling suggested a new expansion origin for the Southern population of *Amborella*. Previous studies based on species distribution modelling did not identify this area as a possible refugium for rainforest plant species [13, 15]. Distribution modelling may be impaired in New Caledonia by lack of accuracy and spatial uncertainty in general circulation models [50]. This southern area, however, overlaps a major centre of present relict angiosperm diversity [15, 51] and could have preserved angiosperm diversity under recent environmental fluctuations. In this regard, our results suggest that demo-genetic modelling can complement species distribution modelling or descriptive phylogeographic analyses, to get insights into the location and dynamics of past refugia.

### Dynamics of post-glacial population range expansion

The shape and speed of range expansion were likely influenced by environmental heterogeneity along with more intrinsic properties of species migration and growth rates [52]. Spatially explicit modelling suggested that the topography could have influenced gene flow among adjacent populations, limiting dispersion at low elevation since ~12,800 years BP. Our posterior estimates on friction coefficients suggest that *Amborella* was less likely to cross low elevations (valleys) than higher ones (mountain ridges). In addition, non-spatial modelling revealed that gene flow was 3-fold higher from Northern to Southern populations than inversely. The Northern population has had a greater and steadier size over the last 23,400 years, suggesting it could thus have been a source population for Southern populations during the expansion phase. The spatial model suggested that present genetic diversity was shaped by at least two waves of expansion stemming from at least two different genetic pools, one located in the neighbourhood of the Aoupinié, the other from the neighborhood of the Dogny plateau in the Southern part of *Amborella* distribution. Based on the spatial coalescent analysis, the Ba population (Fig 1) was first colonized by the Northern wave before being soon reached by the Southern one. This secondary contact zone between Northern and Southern expansion waves probably drove the admixture observed in Ba population along with high heterozygosity and low private allele counts. The Aoupinié could thus have been source population for the expansion with later admixture from the Southern wave.

Local population densities reached habitat carrying capacities in a few generations during the expansion phase for both North and South ancestral populations. The high growth rate and Northern and Central migration rate values indicated low founder effects above 21.3°S latitude. The expansion rate was higher in the Southern zone: the population size increased by 530% compared to its initial population size, indicating high post-glacial demographic expansion. Yet, based on spatially explicit modelling, the carrying capacity and effective migration rate in the zone were lower than in the Central or Northern ones, suggesting stronger genetic drift in the South. These results indicated that *Amborella* density had likely varied in space and time over the last 12,800 years, especially in the Southern zone, where more frequent fire events about 3,000 years BP [42, 49] could have favoured open savannah over lowland rainforests. Low migration rates and topographic heterogeneity in the South likely led to anisotropic range expansion, genetically characterized by an excess of rare alleles in peripheral populations and increased genetic differentiation among populations.

### Insights of demo-genetic models into evolutionary ecology

Using a large set of SNPs and geo-referenced microsatellite data, and by combining two demo-genetic modelling frameworks, we inferred the demographic history, the location of ancestral populations and the subsequent expansion dynamics of *Amborella trichopoda* in New Caledonia. The approach allows addressing past population dynamics in a spatially-explicit way, and it overcomes some limitations of inferring distribution changes from species distribution models (SDMs) neglecting demography and spatial population dynamics [53]. SDM assumptions neglect the influence of genetic variability, extinction-recolonization metapopulation dynamics and range expansions [54], but we suggest that demo-genetic modelling can overcome these limits. We also illustrated that paleo-climate layers from various general circulation models introduced uncertainty in habitat suitability projection, a known issue for insular contexts [43]. Demo-genetic modelling is an alternative and complementary framework to investigate species distribution change. Specifically, they acknowledge the influence of dispersal limitations, here modelled as a two-dimensional stepping-stone model with topography-dependent friction [28]. They however neglect long-distance dispersal processes, which are considered rare, especially in the understory where *Amborella trichopoda* grows, but which could potentially have strong impact on the evolution of genetic diversity [52].

Overall, demo-genetic models offer promising prospects for understanding evolutionary responses of organisms to climate change, especially when they can be compared to reliable SDMs and/or paleo-chorological maps derived from fossil or isotopic records [55]. Comparison of independent datasets and models can provide complementary insights into the relative balance of geographical shifts *versus* ecological niche shifts and into the evolution of genetic diversity under climate change. We argue that since species adaptability depends on current genetic diversity and its historical drivers [54], the critical interplay between climate and genetic variations should be more thoroughly investigated to better predict biodiversity dynamics under the influence of ongoing and future climate change.

## Supporting information

S1 Appendix Bioinformatic data pre-processing. (PDF)

S2 Appendix Data analyses and modelling. (PDF)

S1 Table Genetic sampling design. (PDF)

S2 Table Matrix of pairwise allele sharing distances. (PDF)

S3 Table Typology of the non-spatial demo-genomic models. (PDF)

S4 Table Non spatial demo-genomic model comparison. Maximum composite likelihoods, AICs and evidence ratios for the 13 tested demo-genomic models.
(PDF)

S5 Table Parameter estimation for the most likely demo-genomic model (2M). (PDF)

S6 Table Predictive check of the adjusted 2M demo-genomic model. Genetic summary statistics derived from the observed probabilistic SFS vs. their prediction under the adjusted 2M scenario.
(PDF)

S7 Table Mean-standardized root-mean-square error (SRMSE) of parameter estimation for the spatial coalescent model. (PDF)

S8 Table Mean posterior estimates for key parameters of the spatially explicit demo-genetic model. (PDF)

S1 Fig Genetic structure. (A; C) Inference using the ADMIXTURE software; (B; D) using the sNMF software; (E) using the full-genotype likelihood software NGSAdmix.
(TIF)

S2 Fig Posterior parameter distributions of the spatial demo-genetic inference. Prior densities (grey-filled) and marginal posterior densities (red-filled) for 16 parameters of the spatially explicit demo-genetic modelling. The geographical coordinates of the ancestral populations are the COORD_* parameters.
(TIF)

S3 Fig Posterior predictive check for the spatial demo-genetic modelling. The observed dataset (red cross) represented in the space of the first two principal components computed over the 159 summary statistics from the 2,850 posterior simulations under the adjusted spatially explicit demo-genetic model. The envelope contains 90% of the simulated datasets.
(TIF)

S4 Fig MaxEnt estimated probabilities of *Amborella trichopoda* paleo-occurrence. Maps of paleo-occurrence probabilities during the mid-Holocene (6,000 years BP) and during the Last Glacial Maximum (21,000 BP), based on the simulations of four different global circulation models: CCSM4 (CC), MPI-ESM-P (ME), MIROC-ESM (MR) and IPSL-CM5A-LR (IP). **(A)** Continuous logistic probabilities of paleo-occurrence; **(B)** categories of paleo-occurrence probability, defined using two previously published ROC-based cut-off values; **(C)** same as (A), but following a clamping procedure which down-weighted the probabilities where paleo-climatic conditions fell out of the climatic range represented in the training set at present.
(TIF)
