# Supplementary material for: Two disjunct Pleistocene populations and anisotropic postglacial expansion shaped the current genetic structure of the relict plant Amborella trichopoda
Source: PLoS One. 2017 Aug 18;12(8):e0183412. doi: 10.1371/journal.pone.0183412 (PMC5562301; doi:10.1371/journal.pone.0183412)
Supplement: S1 Appendix — (PDF) [file pone.0183412.s001.pdf]

Appendix S1

1 Bioinformatic data pre-processing 2
1.1 Pipeline A: sequence mapping 3
1.2 Pipeline B: SNP calling 5
1.2.1 SNP dataset for genetic structure analyses 5
1.2.2 List of SNP clusters 5
1.3 Pipeline C: probabilistic joint site frequency spectrum 7
2 Supplementary references 9

1 Bioinformatic data pre-processing

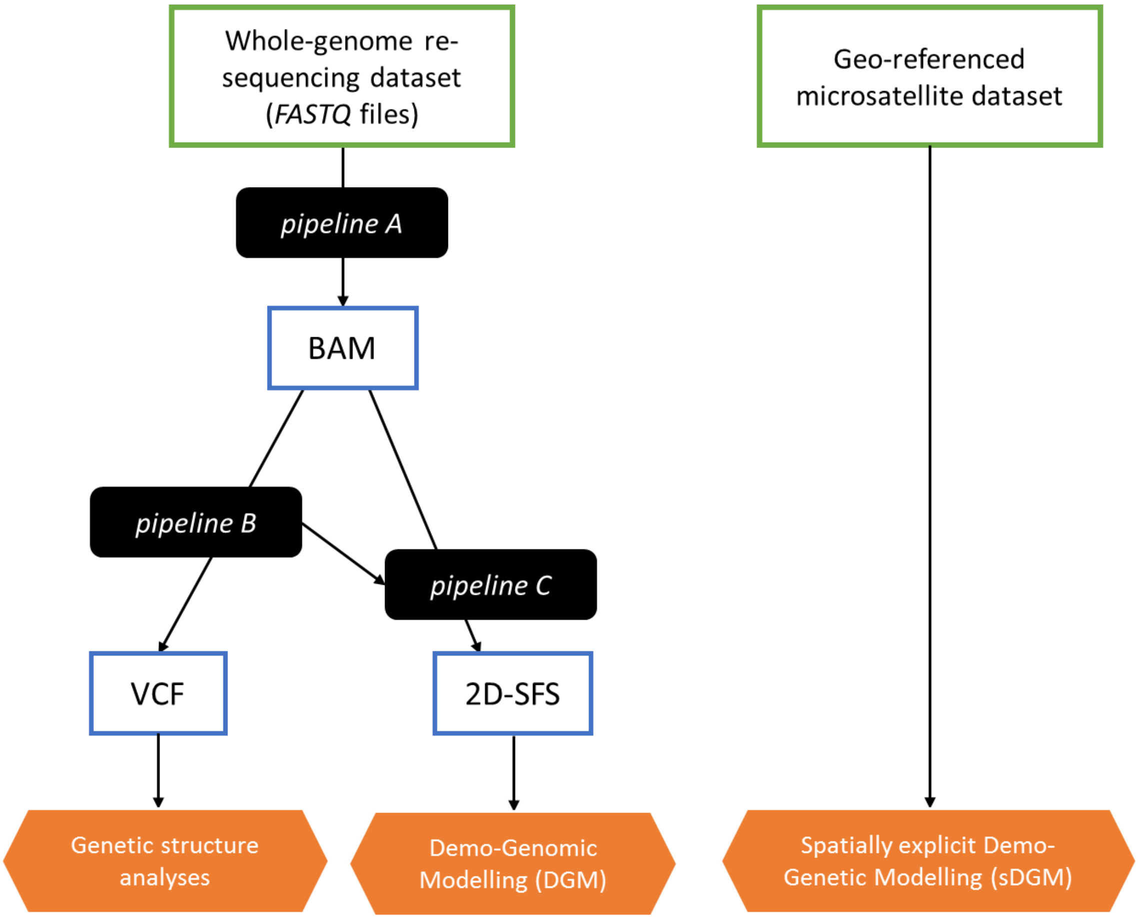

Figure A – Meta-pipeline describing the workflow for genetic data analyses and modelling. Input genetic datasets are represented in green boxes while intermediate bioinformatic files are represented in blue boxes. Orange polygons indicate the specific analyses carried out from each specific files generated using the bioinformatic pipelines (black-filled boxes) described in the following sections.

1.1 Pipeline A: sequence mapping

Whole-genome sequences from the 12 sampled individuals were mapped to the reference genome of *Amborella trichopoda* [1] using the bioinformatic **pipeline A** described below.

For all sampled individuals, paired-end Illumina HiSeq reads were retrieved from the FTP of the Amborella Genome Project [1]. Sanger encoding of the FASTQ files was checked and adaptors were trimmed out using the software cutadapt 1.8 [2]. Reads with a mean sequence quality lower than 30 were discarded. Retained forward and reverse reads were paired using a Perl script [3]. Processed reads were mapped onto the reference genome of *Amborella trichopoda* [1] using BWA 0.7.2 with the *aln* and *sampe* protocol [4]. We used the following mapping parameters: 4% of mismatch at most, 1 open gap at most, mismatch penalty of 3. We then discarded reads which verified any of the following criteria: reads were unmapped, reads had a low mapping quality < 5, read pairs were not properly aligned, and reads were cut in pieces (CIGAR flag badly shaped). Filtered mapped reads were then locally realigned to address problems of INDEL misalignment, using GATK 3.3 IndelRealigner [5] and default parameters. Optical duplicates resulting from PCR biases were finally removed using Picard Tools 1.83 MarkDuplicates.

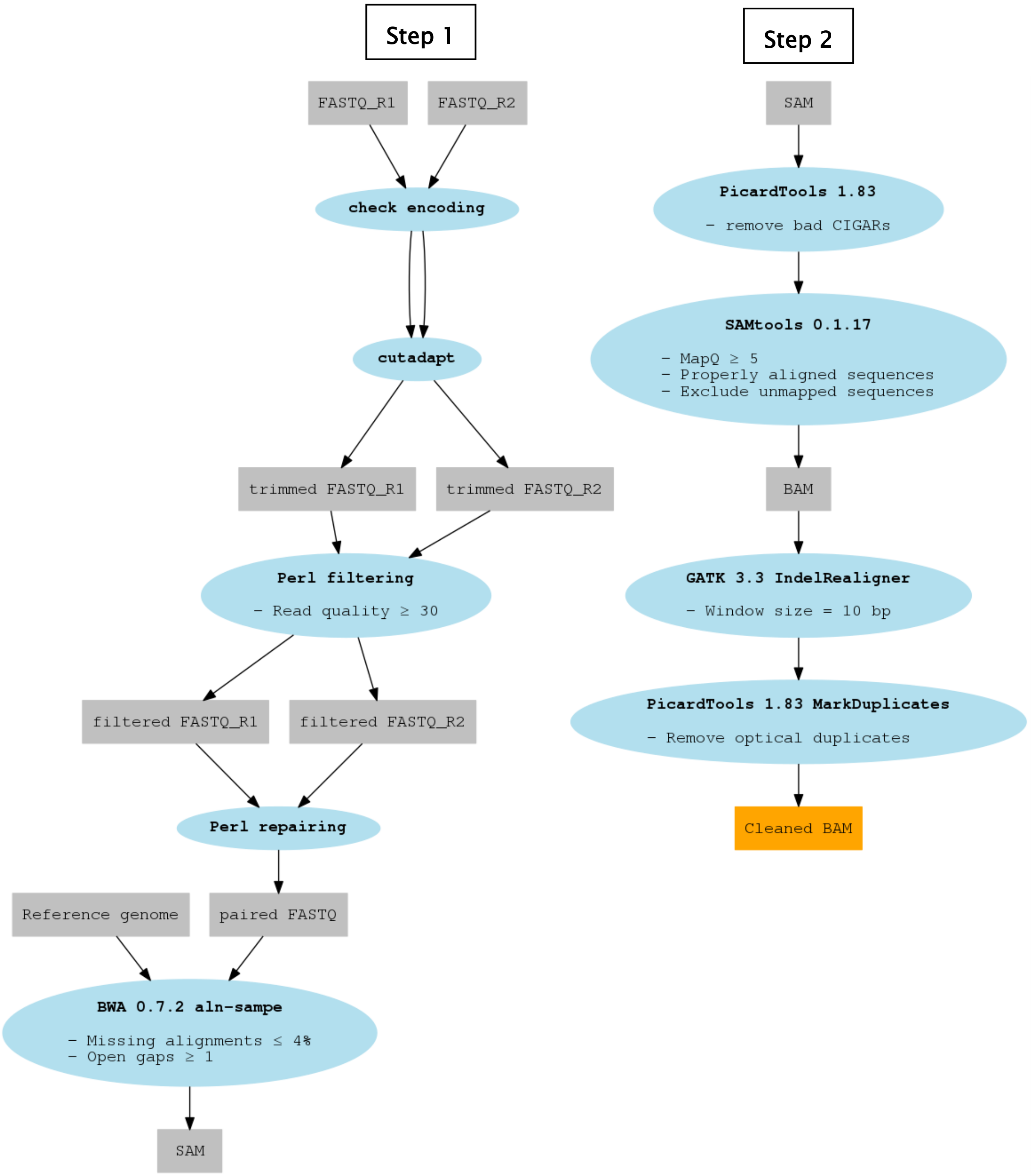

Pipeline A used to process raw paired-end sequences (FASTQ_R1 and FASTQ_R2 files) into mapped sequence files (“Cleaned BAM”). Software packages (including particular modules) are given in blue ellipses, their names in bold fonts and their options specified in a list underneath. Intermediate files are shown as grey boxes. The final pipeline output is shown as an orange box.

1.2 Pipeline B: SNP calling

Based on the BAM files obtained from **pipeline A**, we called genetic variants among the 12 sampled individuals using the GATK 3.3 recommended pipeline: local *de-novo* assemblies of sample haplotypes (*HaplotypeCaller* module) followed by the cross-sample genotyping of called haplotypes (*GenotypeGVCFs* module) [5]. The variants were stored in a raw VCF file containing 7,458,468 biallelic SNPs.

1.2.1 SNP dataset for genetic structure analyses

From the raw VCF file, we generated a dataset of filtered SNPs which were used in genetic structure analyses. The SNPs considered for this final dataset had to meet all of the following conditions:

- strict biallelic single nucleotide polymorphism,
- Phred-scaled quality score for genotype assertion, QUAL > 500,
- No missing genotype across samples,
- Mean genotype quality, GQ_MEAN > 30.0,
- Standard deviation on genotype qualities, GQ_STDDEV < 30.0.

After the filtering procedure, we obtained 333,181 SNPs. We then uniformly sampled, across the genome, 100,000 SNPs from the filtered VCF in order to minimize linkage disequilibrium between markers.

1.2.2 List of SNP clusters

Before scanning whole raw sample genomes to construct the site frequency spectrum (**pipeline C**), we first processed the VCF file to identify a list of clustered SNPs to discard. These SNPs were potential artefacts caused by biased assemblies (related to multigene families, repeated sequences…) in the reference genomes and could therefore bias the SFS. We observed that these artefactual SNPs were characterized by the following specificities:

they were heterozygous for all individuals, had a sequencing depth higher (often ≥ 2-fold) than the mean depth, and were located in dense clusters.

SNPs were considered clustered when there were $n \geq 3$ of them present within any sliding 70 base long window of the genome. They were identified using GATK 3.3 *VariantFiltration* after removal of variants with QUAL < 40.

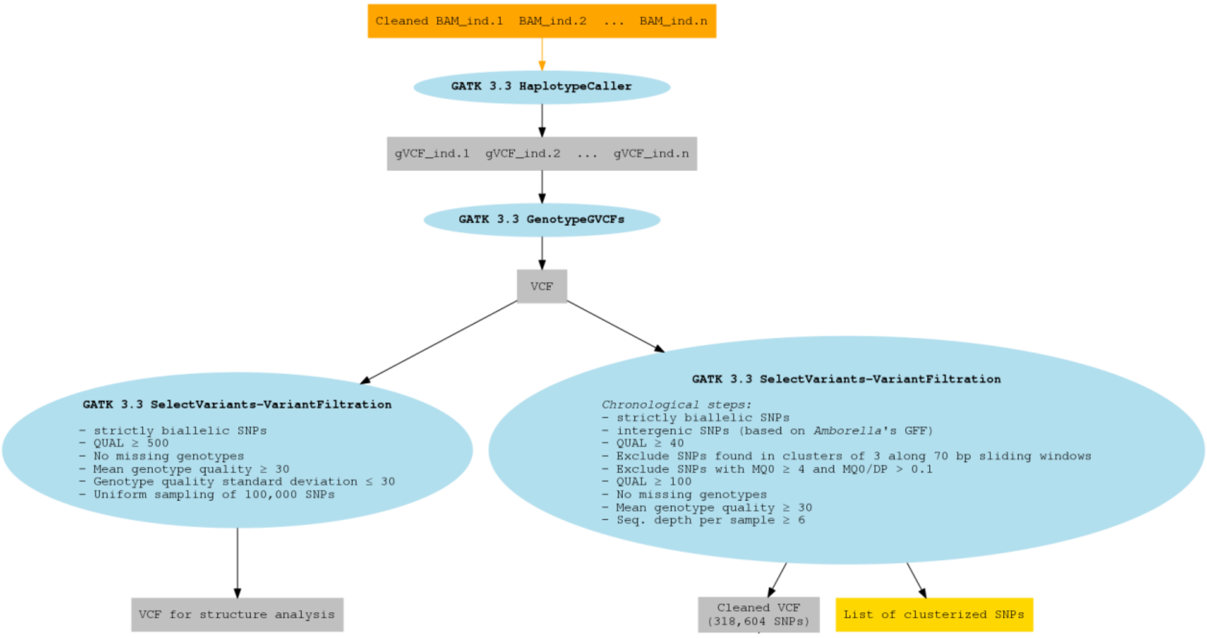

Pipeline B used to process cleaned sample-specific mapped sequence files into a set of single nucleotide polymorphisms used for genetic clustering analyses (left side) and into a temporary list of clustered SNPs passed to the next **pipeline C** (right side).

1.3 Pipeline C: probabilistic joint site frequency spectrum

Based on the 11 individuals (without *Aoupinié*) clustering at K=2, we summarized whole-genome polymorphisms into a two-dimensional joint and folded site frequency spectrum (SFS). An SFS summarizes the full polymorphism information from independent genetic markers and is accessible to likelihood-based demo-genomic inferences. Here, the 2D-SFS is a matrix specifying the number of SNPs found at the coordinates (f_N,f_S) of minor allele frequencies (or counts) in both groups *North* and *South*.

The SFS is sensitive to the sequencing error rate and to the sequencing depth at each position of the reference genome (*i.e.* the number of reads available at each position of the reference genome) which alter the power of genotype inference. If the coverage depth is low, usual methods based on genotype calling (*e.g.* VCF files) fail to recover the true amount of SNPs with very low minor allele frequencies, potentially leading to significant biases in demographic inferences.

Indeed, the lower the coverage depth in the whole-genome sequence dataset, the more uncertain the inference of individual genotypes is. To summarize whole-genome polymorphisms by taking genotype likelihoods into account, we therefore constructed a probabilistic site frequency spectrum which we then used for demo-genomic inferences [6].

Based on the BAM file output from **pipeline A**, the SFS was computed using ANGSD 0.9 [7]. Genotype likelihoods were computed using the SOAPsnp sequencing error model which is based on calibration matrices of sequencing errors computed in a first pass across the genome [8]. Based on calibration matrices, we then computed the SFS (polarizing allele state on the reference genome) at the positions of the reference genome which met the following criteria:

- for all BAMs, reads containing the position are uniquely mapped,
- the position is located in an intergenic region (based on *A. trichopoda* genomic annotation GFF file and assuming that intergenic SNPs are less likely under selection than coding ones),
- for all BAMs, individual depth $\geq$ 6 at the position,

- for all BAMs, sequence quality ≥ 35 at the position,
- for all BAMs, mapping quality ≥ 25 at the position,
- across BAMs, not too many different alleles at the position (-*setMaxDiffObs*=1),
- the position is not referenced in the list of clustered SNPs (as obtained from **pipeline B**, yellow box on the right).

In the absence of any close living relative for *A. trichopoda*, we cannot determine the ancestral and derived states of biallelic SNPs. In such cases, the SFS cannot be based on *derived* allele frequencies but rather on *minor* allele frequencies. For a given SNP, the minor allele frequency is defined as the frequency of the least frequent allele across all sampled individuals. In a first step, allelic polymorphisms were polarized according to the reference base considered as the (pseudo) ancestral allele. This procedure generated a (pseudo) derived 2D-SFS. We then *a posteriori* folded it, *i.e.* converted *derived* allele counts (or frequencies) into *minor* allele counts (or frequencies). Using simulations, we checked that this *post*-folding procedure was not introducing any biases in the SFS folding. Note that in situations where we cannot discriminate between the minor/major alleles of a SNP (because the frequency of both alleles is ½ across sampled individuals), we recorded a half-count in the complementary cells of the SFS for each allele alternatively considered as minor (as in fastsimcoal).

At the end of the analysis, the probabilistic 2D-SFS contained 118,907 SNPs.

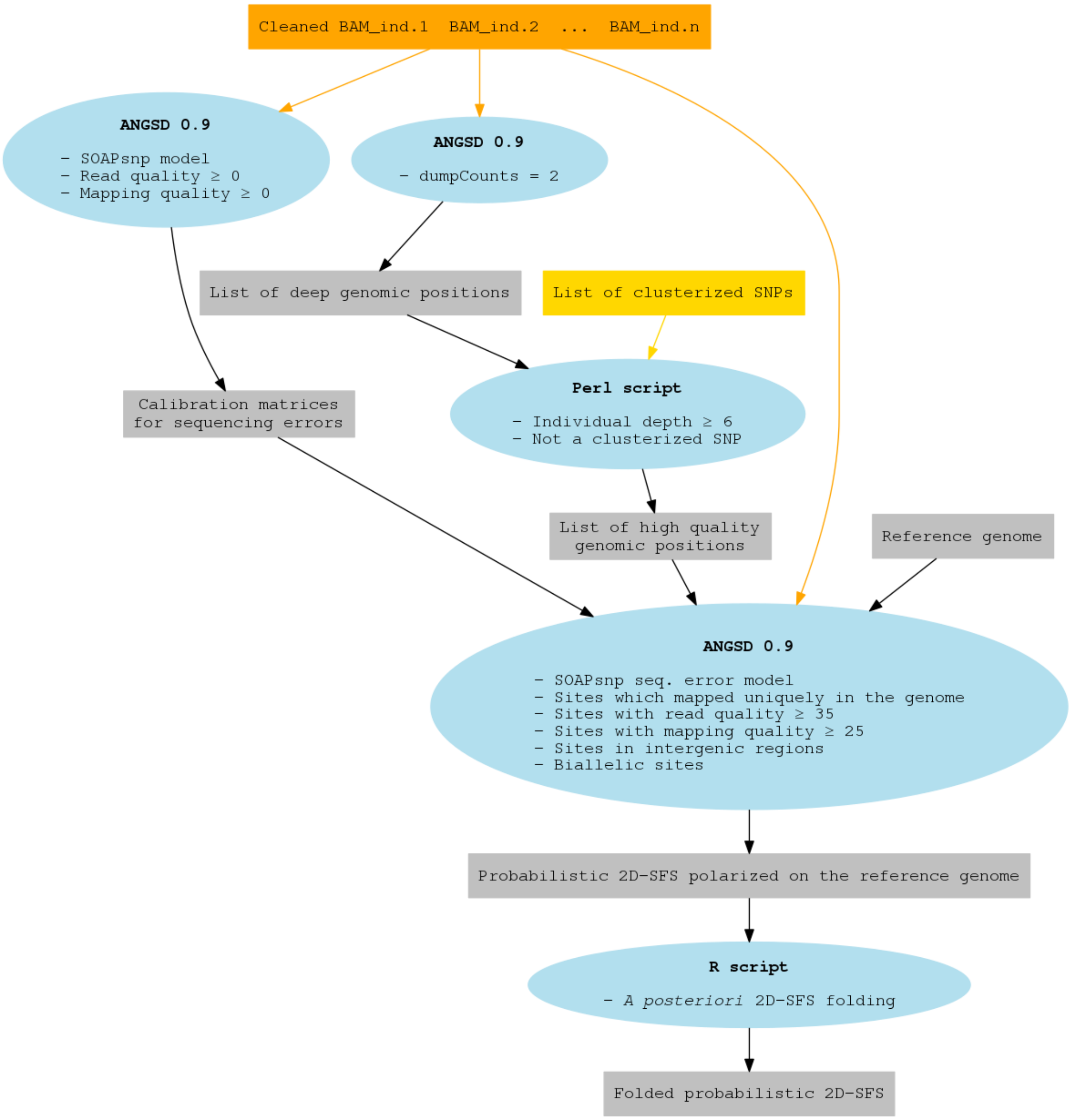

Pipeline C used to process mapped reads from the 11 sampled individuals into a folded probabilistic joint site frequency spectrum. The yellow box is the list of clustered SNPs output from the previous **pipeline B**.

2 Supplementary references

1. Amborella Genome Project (2013) The Amborella genome and the evolution of flowering plants. Science 342.

2. Martin M (2011) Cutadapt removes adapter sequences from high-throughput sequencing reads. 2011 17.

3. Nabholz B, Sarah G, Sabot F, Ruiz M, Adam H, et al. (2014) Transcriptome population genomics reveals severe bottleneck and domestication cost in the African rice (Oryza glaberrima). Molecular Ecology 23: 2210-2227.

4. Li H, Durbin R (2009) Fast and accurate short read alignment with Burrows-Wheeler transform. Bioinformatics 25: 1754-1760.

5. McKenna A, Hanna M, Banks E, Sivachenko A, Cibulskis K, et al. (2010) The Genome Analysis Toolkit: a MapReduce framework for analyzing next-generation DNA sequencing data. Genome Res 20: 1297-1303.

6. Excoffier L, Foll M (2011) fastsimcoal: a continuous-time coalescent simulator of genomic diversity under arbitrarily complex evolutionary scenarios. Bioinformatics.

7. Korneliussen T, Albrechtsen A, Nielsen R (2014) ANGSD: Analysis of Next Generation Sequencing Data. BMC Bioinformatics 15: 356.

8. Li R, Li Y, Fang X, Yang H, Wang J, et al. (2009) SNP detection for massively parallel whole-genome resequencing. Genome Res 19: 1124-1132.
