# Supplementary material for: Two disjunct Pleistocene populations and anisotropic postglacial expansion shaped the current genetic structure of the relict plant Amborella trichopoda
Source: PLoS One. 2017 Aug 18;12(8):e0183412. doi: 10.1371/journal.pone.0183412 (PMC5562301; doi:10.1371/journal.pone.0183412)
Supplement: S2 Appendix — (PDF) [file pone.0183412.s002.pdf]

Appendix S2

1 Data analyses and modelling 2
1.1 Genetic diversity analyses 2
1.1.1 Genetic structure 2
1.1.2 Differentiation indices and pairwise genetic distances 3
1.2 Non-spatial demo-genomic coalescent modelling 4
1.2.1 Model design 4
1.2.2 Prior search ranges 6
1.2.3 Model comparison 7
1.2.4 Estimation precision 7
1.2.5 Model validation 8
1.2.6 Model with more than two ancestral populations 8
1.2.7 Structured model 10
1.3 Spatially explicit demo-genetic coalescent modelling 12
1.3.1 Description of the demo-genetic coalescent model 12
1.3.2 Log-bilinear transformation of friction coefficients from a digital elevation raster 15
1.3.3 Statistical inferences 16
1.3.4 Accuracy of parametric estimation 17
1.3.5 Posterior predictive check 17
1.3.6 Test of robustness for the inferred locations of expansion origins 18
1.4 Species Distribution Modelling (SDM) 23
1.4.1 Discretization of paleo-occurrence probabilities 23
2 Supplementary references 24

1 Data analyses and modelling

1.1 Genetic diversity analyses

1.1.1 Genetic structure

Genetic structure analysis was performed using two unsupervised algorithms handling large SNP datasets:

(1) sNMF [1], a model-free algorithm with a least-square optimization based on factorization of the genotype matrix into a matrix *Q* of individual ancestry coefficients and a matrix *G* of ancestry allele frequencies,

(2) ADMIXTURE [2], a model-based algorithm.

For the analyses, we used 100,000 filtered SNPs output from the **pipeline B**. To estimate the most probable number of genetic clusters, we varied the prior number of clusters (*K*) from 1 to 10 for both software programs.

For sNMF, we performed 20 runs per *K* value with 200 iterations per run. The regularization parameter was set at 10 [1]. Cross-validation based on cross-entropy criteria for each run per *K* was computed with a 5% fraction of masked genotypes.

For ADMIXTURE, we used default parameters and computed a cross-validation error for each *K* using 20 cross-validations.

We defined the most probable number of cluster(s) (*K*) as the one minimizing both cross-entropy criterion (sNMF) and cross-validation error (ADMIXTURE).

Owing to potential biases entailed by low-coverage sequencing, an independent clustering analysis was realized with NGSAdmix (Skotte et al. 2013) based on the full genotype likelihoods computed in ANGSD 0.9 with the SAMTools sequencing error model (Skotte et al. 2013). Called positions were filtered on quality scores, minor allele frequencies and sample coverage, leaving a subset of 10^7 sites.

1.1.2 Differentiation indices and pairwise genetic distances

Based on the VCF dataset previously used for structure analysis and after removal of the Aoupinié individual, Weir and Cockerham [3] weighted F_{ST} was calculated between the two major genetic groups using VCFTools 0.1.13 [4]. The significance of the F_{ST} was estimated by performing 999 permutations of sample assignation to the genetic clusters, the null hypothesis being that all individuals are sampled from a panmictic population. An empirical *p*-value was then calculated as the ratio between the number of permuted F_{ST} values equal or superior to the observed F_{ST} value. Pairwise allele sharing distances between individuals [5] were also estimated, using a custom R script.

1.2 Non-spatial demo-genomic coalescent modelling

1.2.1 Model design

We compared a set of 13 demographic scenarios (see the table below)—assuming either a single or two ancestral population(s), out of which the two currently major genetic populations were derived. In addition to the number and size of putative refugial populations, the 13 demographic scenarios also differ in the specification of the age of divergence between the two major genetic groups relative to the contraction phase. We also considered variations in the recent demographic dynamics: (a) abrupt or smooth population growth, and (b) presence or absence of gene flows.

Demo-genomic models are simulated using the Kingman coalescent based on a Wright-Fisher population model, assuming an infinite-allele mutation model.

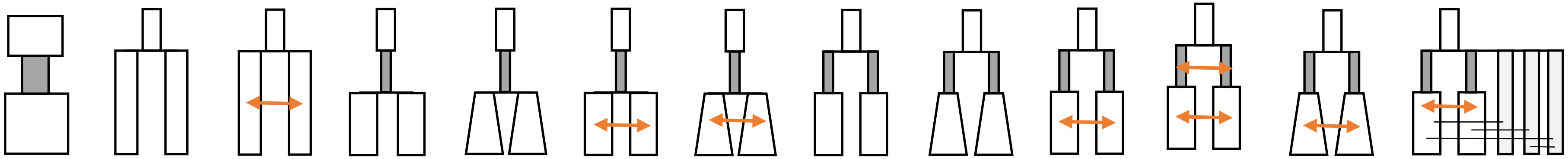

	Po	Do	DoM	1	1G	1M	1MG	2	2G	2M	2MM	2MG	3I
Number of expansion origins	1	0	0	1	1	1	1	2	2	2	2	2	2
Recent gene flow	No	No	Yes	No	No	Yes	Yes	No	No	Yes	Yes	Yes	Yes
Gene flow between ancestral populations	No	No	No	No	No	No	No	No	No	No	Yes	No	No
Exponential expansion	No	No	No	No	Yes	No	Yes	No	Yes	No	No	Yes	No

1.2.2 Prior search ranges

Historical events are defined forward in time.

Parameter	Distrib.	Lower bound	Upper bound	Notes
Present population sizes	U	10	400,000	For all models except Po, there are two independent present population sizes.
Age of bottleneck onset[b] (*Tb*)	U	5	200,000	Corresponds also to the age of divergence for models 2xx.
Age of expansion onset[b] (*Te*)	U	5	200,000	Corresponds also the age of divergence for models 1xx.
Neutral substitution rate	U	5.00E-09	1.00E-07	
Early population size	U	100	400,000	
Migration rates[c]	LU	1.00E-05	0.05	For all models, we set the possibility of asymmetric gene flow (= 2 independent migration rates parameters).
Bottlenecked population ratio	U	1.00E-04	1	For models 2xx, there are two “bottlenecked” population ratios. For models 1xx, Do and PoB: only one ratio.
Population sizes at expansion onset	U	1.00E+01	400,000	For models with continuous expansion growth.

[a] “U” stands for “uniform” and “LU” for log_{10}-uniform distributions.

[b] Due to the existence of two likelihood attractors for model 2M, one with unrealistic divergence times, *Tb* and *Te* search ranges were restricted to 5-25,000 and 5-50,000 respectively. Ages are specified in generations before present.

[c] For 1M, 1MG, DoM upper bound = 1E-3 to reduce computation time

1.2.3 Model comparison

To compare demographic scenarios and estimate their parameters, we used the composite likelihood maximization approach implemented in *fastsimcoal2* [6]. This approach is based on the comparison of the composite likelihoods (CL) of model-based SFSs, given the observed SFS. Note that we filled in the cell [0,0] of the SFS (corresponding to the monomorphic bases across populations) to obtain better estimates of θ.

To maximise the CL, *fastsimcoal2* performs an Expectation-Conditional Maximisation (ECM) algorithm where each parameter is optimized sequentially, keeping other parameters at their previous values [6]. CL estimates can be sensitive to the initial conditions, so we performed 120 independent estimation replicates using various parametric seeds. For each estimation, we ran a series of 10 to 70 ECM cycles stopping the convergence when the likelihood weight decay fell below 10^{-2}. We simulated 250,000 to 700,000 coalescent trees per likelihood computation. Convergence was checked for each model *a posteriori*. For each model, we retained the one run with overall maximum CL among the 120 runs performed. Runs which converged to unrealistic parametric values (any island with an effective size estimated below 120 haploid individuals during more than 5,000 generations) were excluded from the comparison.

Since the distribution of the composite likelihood ratio test is often unknown [6] and since models differ in their degrees of freedom, we used the Akaike Information Criterion (AIC) as a way to compare model fits.

1.2.4 Estimation precision

For the fittest scenario, we estimated confidence intervals around the point estimate for each parameter by conducting parametric bootstrapping. We simulated 100 SFSs out of the fittest adjusted scenario and subsequently used them as pseudo-observed datasets. For each pseudo-observed SFS, we performed 20 parametric estimation runs out of which we extracted the parameter values of the overall fittest run. The sequence of the 100 fittest parametric values obtained for the 100 pseudo-observed SFSs were used to compute the empirical 95% confidence intervals for each parameter.

1.2.5 Model validation

The predictive power of the retained model with its maximum likelihood point estimates was assessed by generating DNA polymorphisms along 105 chromosomes of 300 bp each, corresponding to the total number of filtered sites analysed to infer the joint 2D-SFS. Observed and predicted polymorphisms were summarized into six statistics using a custom R script: number of segregating sites, proportion of singletons, of private and shared alleles, expected heterozygosity and Wright's F_{ST}. Results are given in S6 Table.

1.2.6 Model with more than two ancestral populations

To test whether our sampling sizes allowed to correctly identify a model with more than two ancestral populations from a model assuming exactly two, we generated 50 pseudo-observed datasets (PODs = folded 2D-SFS) using the adjusted 2M model and 50 PODs under a 3M model. The 3M model is an extension of the 2M model assuming two isolated bottlenecked populations for the North lineage, instead of one only. The 3M model therefore assumes a total of 3 bottlenecked populations (2 for the North + 1 for the South). The (i) effective size of the additional bottlenecked population and (ii) the fraction of lineages of the North present population which originated from this additional population were set as random parameters. A model selection (2M vs 3M model) was then performed for each of the 100 PODs based on AIC criterion.

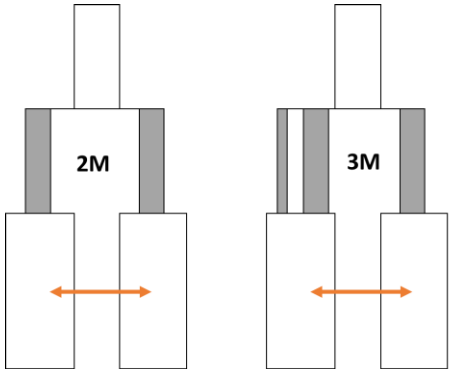

Models 2M and 3M

Results. If we consider the "2M" as the positive outcome, the false negative rate is **FNR** = 27/(23+27) = **54%** and the false positive rate is **FPR** = 22/(22+28) = **44%.**

Confusion matrix

		Estimated as	
		**2M**	**3M**
Simulated as	**2M**	23	27
	**3M**	22	28

This complementary analysis suggests that a model assuming three ancestral populations is not identifiable from a model assuming two ancestral populations.

1.2.7 Structured model

To assess the impact of population structure on the estimation of population size changes [7,8], we implemented a **3I** model (an extension of the 2M model) representing a 5-island metapopulation model with 3 unsampled islands (filled in green in the figure below) of size N_{Ghost}. These unsampled islands were allowed to exchange between themselves and with the 2 sampled islands *m* (symmetric) migrants per generation after (forward) the contraction (grey-filled) period. A series of 120 independent runs of composite likelihood maximization were performed under the same procedure as described in the Model Comparison section.

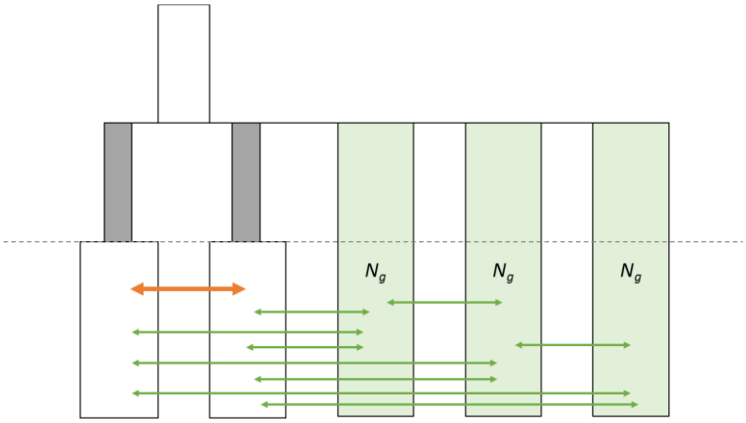

Model **3I**

Results. Among the 120 runs, only three have an AIC inferior to the best-fit 2M model presented in the main text. Those runs are described in the following table, in columns. There is no biological reason to reject the adequacy of these three 3I run estimates, yet we might probably face the same identifiability issue as with the 3 ancestral population model (3M, aforementioned). Point estimates for parameters of interest, like the age of expansion onset, are close to the ones obtained from the 2M model. The point estimate for the age of divergence is however 3 to 6-fold higher than under the 2M model. The ratio [present/ancestral population sizes] for the North is comparable to the 2M model (= 1 in 3I and 2M models) but slightly lower for the South (2.5; 2.7; 4.4 for the 3I runs *vs*. 6.6 for the 2M best run).

Table – Maximum composite likelihood estimates of the 3 best runs for the 3I structured model.

	**Run 1**	**Run 2**	**Run 3**
Maximum Estimated Ln-Likelihod	-1168179.632	-1168201.882	-1168221.755
Ne North	55192	28173	31025
Ne South	71212	31589	35126
Ne Ghost	13076	20018	13094
Ne Ancestral North	54622.84906	27638.42014	30586.04899
Ne Ancestral South	27501.19849	11496.14055	8010.779358
Ne Pre-divergence	86423	53813	51876
Age of expansion onset (generations)	5049	3868	9387
	~20,000 yBP	~15,500 yBP	~37,500 yBP
Age of divergence (generations)	35918	19318	29741
	~144,000 yBP	~77,000 yBP	~120,000 yBP
Migration North->South	1.25819E-05	1.23277E-05	1.15973E-05
Migration North<-South	1.16483E-05	1.24861E-05	1.23545E-05
Migration<->Ghost	1.12027E-05	2.22179E-05	1.14077E-05
Mutation Rate	5.15842E-09	8.6307E-09	8.07783E-09

1.3 Spatially explicit demo-genetic coalescent modelling

1.3.1 Description of the demo-genetic coalescent model

In this section, we describe the demographic model associated with the spatially explicit demo-genetic modelling (software SPLATCHE 2.01 Ray et al. [9]). This demographic model assumes that an earliest population diverged 5,900 generations before present (BP) into two daughter populations with respective sizes N_1 and N_2 haploid individuals. The species started expanding at 3,200 generations BP. Time estimates are derived from the non-spatial demographic inference but we tested the robustness of our results to the variations of their value.

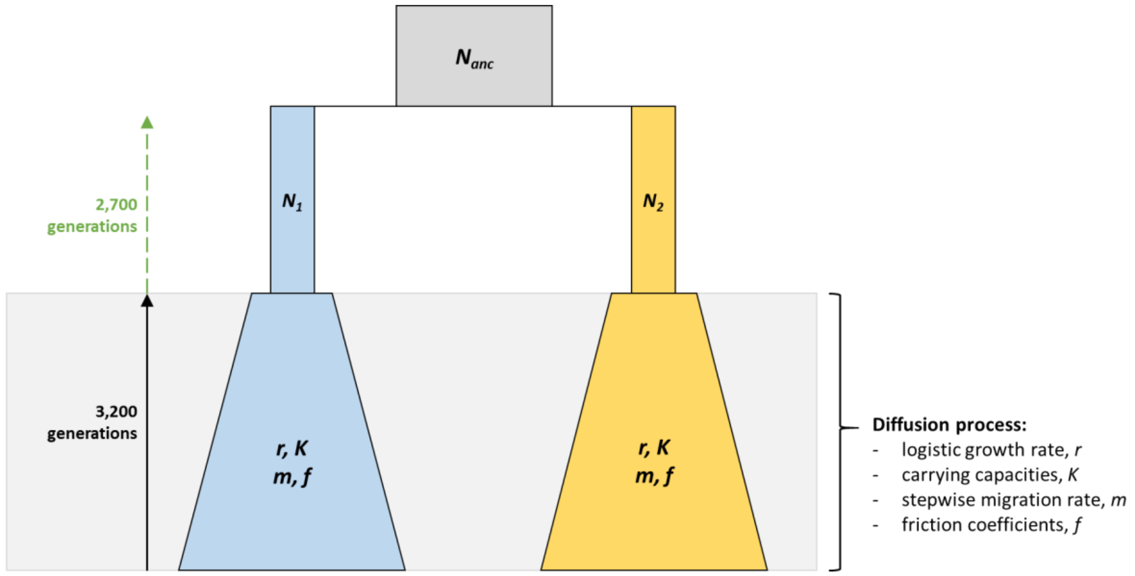

Figure – Illustration of the demographic model associated with the spatially explicit demo-genetic model.

Since 3,200 generations BP, these populations have been expanding and their local population density has been logistically regulated with an intrinsic growth rate r and a carrying capacity K_i. We consider that carrying capacities can have different independent values between three pre-defined zones latitudinally subdividing *Grande Terre*: a Northern zone above latitude 7,668,351 UTM-58S (with a carrying capacity noted K_N); a Central zone between latitudes 7,668,351 and 7,646,970 (K_C); a Southern zone below latitude 7,646,970 (K_S). Besides, since *Amborella trichopoda* is restricted to volcano-sedimentary substrates, we

set the carrying capacities of non-volcanosedimentary soil types of *Grande Terre* at a value of 50 haploid individuals.

Since 3,200 generations BP, adjacent populations have sent migrants with a mean stepwise migration rate m and the proportion of emigrants in each cardinal direction has been controlled by topography-dependent friction coefficients f. To allow greater flexibility in the modelling of landscape-dependent resistance to gene flows, we set a log-bilinear relationship between friction coefficients and elevation (see next section): frictions can vary more or less significantly as a function of the elevation and in independent magnitudes whether the elevations are inferior or superior to an elevation threshold which is defined as a random variable.

The genetic mutation model is a generalized stepwise model for microsatellites (GSM, Kimmel and Chakraborty [10]) incorporating two parameters: μ, the raw mutation rate per base per generation and α, the shape of the gamma distribution characterizing the probability law for the transition scale of microsatellite repeats at each mutational event.

Bayesian prior distributions for demographic parameters mentioned in the figure are summarized in the following **Table A** hereunder.

Table A – Bayesian prior distributions for demographic parameters used in the spatially explicit coalescent modelling.

Parameter	Definition	Prior distribution function	Lower bound	Upper bound
**Forward expansion modelling**				
GROWTHRATE, r	Logistic intrinsic growth rate	uniform	10^{-3}	10
MIGRATIONRATE, m	Stepwise intrinsic migration rate	log-uniform	10^{-7}	1
SOURCE1.Ne, N_1	Effective size of the ancestral population 1	uniform	100	20,000
SOURCE2.Ne, N_2	Effective size of the ancestral population 2	uniform	100	50,000
CARCAP1, K_N	Carrying capacity in the Northern zone	uniform	500	500,000
CARCAP2, K_C	Carrying capacity in the Central zone	uniform	500	500,000
CARCAP3, K_S	Carrying capacity in the Southern zone	log-uniform	500	500,000
MID.ALT, A	Altitudinal threshold for the log-bilinear relationship	uniform	1	1,427
PLAIN.F.COEF, f_p	Friction coefficient of the lowest elevation	$= x^y$ with x ~ $U(1,10^5)$ and $y \in [-1;0;1]$		
SUMMIT.F.COEF, f_s	Friction coefficient of the highest elevation	$= x^y$ with x ~ $U(1,10^5)$ and $y \in [-1;0;1]$		
COORD_1	Longitude/Latitude coordinates of the ancestral population 1	Randomly sampled among non-ultramafic pixels		
COORD_2	Longitude/Latitude coordinate of the ancestral population 2 (with constraint $Lat_2 \geq Lat_1$)*	Randomly sampled among non-ultramafic pixels		
**Coalescent genetic modelling**				
MUTRATE, μ	Mean mutation rate	log-uniform	10^{-5}	$5 \bullet 10^{-3}$
GAMMA	Microsatellite repeat gamma rate (generalized stepwise model)	uniform	0.01	1

* The geographical system used is the UTM 58S, thus latitude increases towards the north. The constraint therefore implies that population 2 will always be north of population 1.

1.3.2 Log-bilinear transformation of friction coefficients from a digital elevation raster

This section details the log-bilinear transformation we applied to the Digital Elevation Map (DEM) of New Caledonia for each simulation to obtain a pattern of resistance to gene flows between neighbouring demes. Following SPLATCHE 2.01 conventions, friction coefficients are bounded by 0 and 1 and a friction close to 1 reflects a higher resistance to gene flow [9].

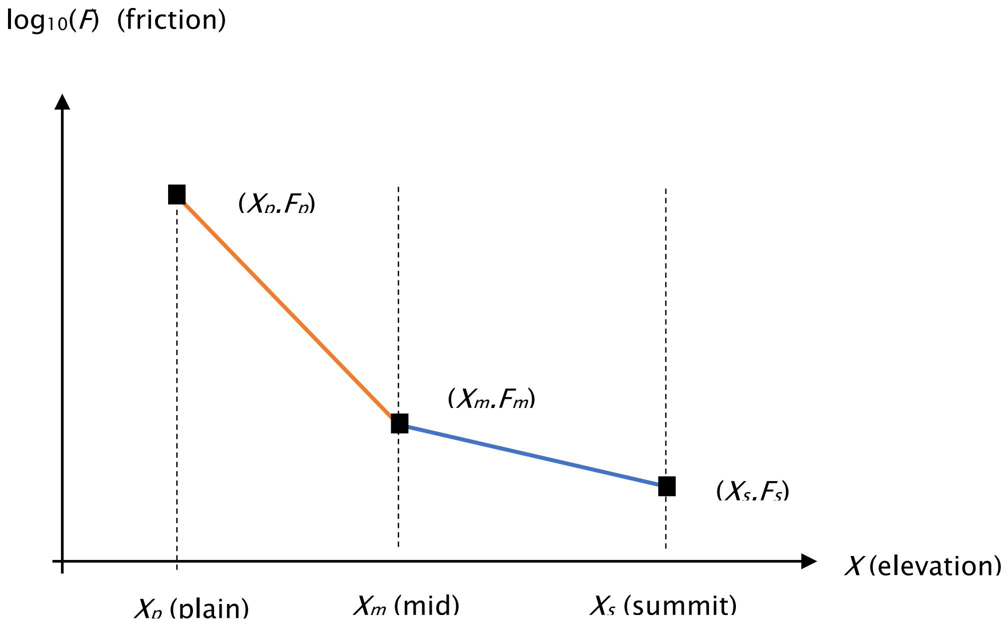

Figure - Bilinear relationship between friction coefficients and elevation.

Defining:

X_p, the lowest elevation (fixed; 0 m)

X_s, the highest elevation (fixed; 1,427 m),

X_m, the mid-elevation, a random variable: $X_m \sim U(1; 1{,}427)$.

The $\log_{10}$-bilinear relationship is defined as:

$$F(X) = \begin{cases} 10^{\left(\frac{\log_{10} F_m - \log_{10} F_p}{X_m - X_p} \cdot (X - X_p) + \log_{10} F_m\right)} & if\ X \leq X_m \\ 10^{\left(\frac{\log_{10} F_s - \log_{10} F_m}{X_s - X_m} \cdot (X - X_m) + \log_{10} F_m\right)} & if\ X > X_m \end{cases}$$

The probability that at generation t, M individuals emigrate from a focal deme to the neighbouring deme i (for sake of simplicity in the explanation, we consider two possible neighbouring demes) is p_i and following SPLATCHE 2.01 conventions:

$$p_i = \frac{1}{f_i \sum_{j=1}^{2} {}^{1}/_{f_j}}$$

With f_i the friction of the neighbouring deme (or pixel) i and j the possible demes neighbouring the focal deme.

If we consider a focal deme with elevation X and two neighbouring demes with respective elevations $X-\Delta X$ and $X+\Delta X$ (with $\Delta X > 0$), the ratio of directional probabilities between both neighbouring demes gives *the proportion of* m *emigrants which will be sent more likely* ΔX *m down* X *than* ΔX *above.*

$$D = \frac{p_{X-\Delta X}}{p_{X+\Delta X}}$$

From the previous equation, with f_X denoting the friction coefficient at elevation X, D simplifies into:

$$\boxed{D = \frac{f_{X+\Delta X}}{f_{X-\Delta X}}}$$

1.3.3 Statistical inferences

The estimation of sDGM parameters was conducted using 600,000 simulations in an Approximate Bayesian Computation (ABC) framework. For each simulation, parameter values are drawn from a prior distribution. Simulations produce individual genotypes that we summarized by a set of 159 summary statistics using a custom R script (see Table B below for further details on the implemented statistics). The ABC procedure approximates the likelihood of the parameters by retaining a minute fraction of the whole simulated datasets which are the Euclidian closest to the observed (here, 0.5% of the full dataset, corresponding to 3,000 accepted simulations) based on the standardized summary statistics. This fraction of accepted simulated datasets provides the Bayesian posterior distribution of the parameters [11]. Since the ABC procedure is sensitive to the curse of dimensionality [12], we performed a dimensionality reduction using neural networks as implemented in the *abc*

package [13] (200 called networks, 20 units in the hidden layer, maximum of 500 iterations per network with a weight decay randomly sampled at 10^{-4}, 10^{-3} and 10^{-2}). Parameters were *logit* transformed, except for the intrinsic growth rate, r, and microsatellite mutation rate, μ, since we assumed that their posterior distribution could possibly depart from their prior range.

Table B – Typology of the 159 genetic summary statistics computed for each microsatellite polymorphism simulated from the spatially explicit demo-genetic model.

	Across loci	Averaged over loci	Per population	Over populations	Pairwise comparison
Allelic richness, A_E	yes	yes	yes	yes	no
Microsatellite repeat range, R	no	yes	yes	yes	no
Expected heterozygosity, H_e	no	yes	yes	yes	no
Garza-Williamson's M	no	yes	yes	no	no
Goldstein's $(\delta\mu)^2$	no	yes	no	no	yes
F_{ST}	no	yes	no	yes	yes

1.3.4 Accuracy of parametric estimation

We evaluated the parameter estimation accuracy around the previously estimated parameter values in ABC by performing 100 local leave-one-out cross-validations. We used the same ABC procedure as above, however, to limit computation burden, we reduced the number of neural networks to 50 and the number of units in the hidden layer to 10. To evaluate the discrepancy between the true parameter values and their mean posterior estimates, we computed the mean-standardized root-mean-square error (SRMSE) and the great-circle distance for the geographical parameters (locations).

1.3.5 Posterior predictive check

To further assess whether the posterior model reproduced genetic datasets close to the observed one, we performed a predictive model check by simulating 2,850 datasets from the fitted model using the adjusted posterior distributions for each parameter. We performed a goodness-of-fit test by assessing whether each observed summary statistic fell within the range of the posterior predictive distribution. To do so, we computed an empirical one-sided

p-value for each summary statistic, *i.e.* the fraction of predictive values that are above the observed value (or below, if the observed value lay on the left side of the predictive distribution). To estimate the number of observed summary statistics which falls within the corresponding posterior predictive distributions, we computed the fraction of summary statistics which have a *p*-value superior to $\alpha = 5\%$.

1.3.6 Test of robustness for the inferred locations of expansion origins

1.3.6.1 Robustness to the age of expansion onset (T_i) and of divergence (T_d)

Method. Based on the posterior adjusted model, we relaxed the age of expansion onset $T_e \sim \log_{10} U\,(150; 10^4)$ and the divergence age as $T_d \sim T_e + \log_{10} U\,(125; 10^4)$ (times are given in generation). We generated 200 pseudo-observed datasets (PODs) for ABC cross-validations (same procedure as for the aforementioned LOOCV).

Results.

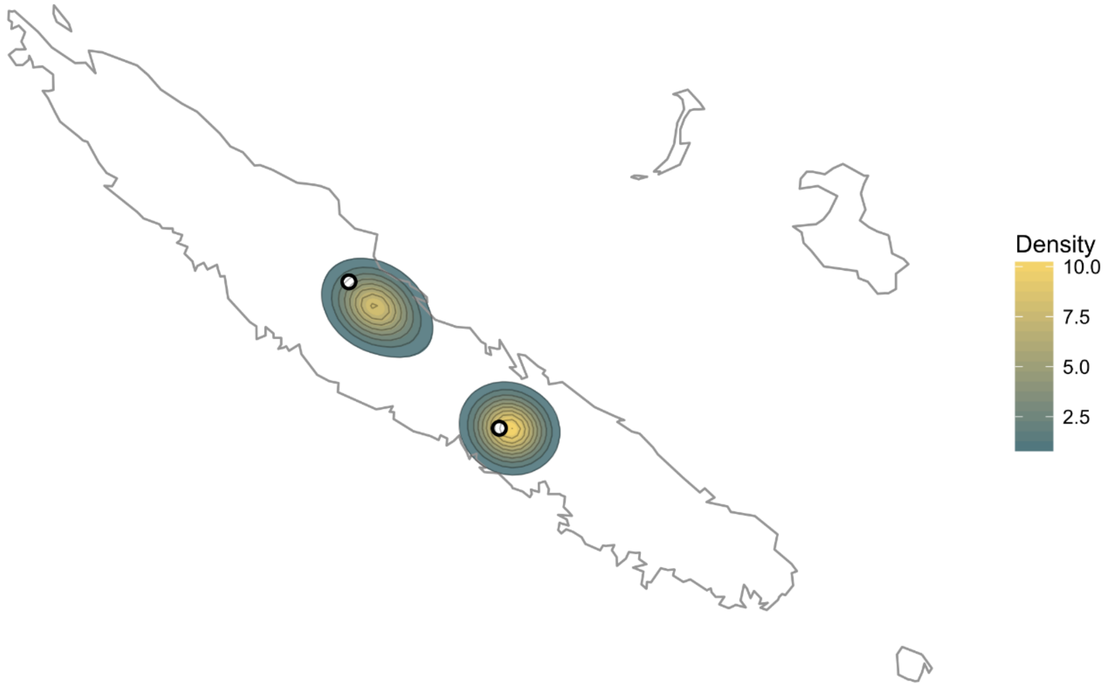

Posterior density of the expansion origins. White dots represent the true locations of expansion origins.

Table - Great-circle error distances (GCED, in kilometres) between true and estimated locations of South and North expansion origins, estimated with 200 cross-validations.

	GCED South (km)	GCED North (km)
**Mean**	6.25	16.77
**Median**	5.53	15.64
**Standard Dev**	7.08	9.29

Conclusion. We are able to recover the locations of the expansion origins whatever the age of expansion onset or the duration of the pre-expansion bottleneck period (within their prior ranges). The estimation of the expansion origin coordinates seems robust to the variation of these two temporal parameters.

1.3.6.2 Robustness to the delimitation of the carrying capacity zones

Method. Based on the posterior adjusted model, we tested the impact that carrying capacity delineation could entail on the inference, by setting $K_N = K_C = K_S$ with $K_N \sim \log_{10} U(100; 5 \cdot 10^5)$. This model assumes therefore a complete homogeneity of the carrying capacities across New Caledonia. We generated 200 pseudo-observed datasets (PODs) for ABC cross-validations (same procedure as for the aforementioned LOOCV).

Results.

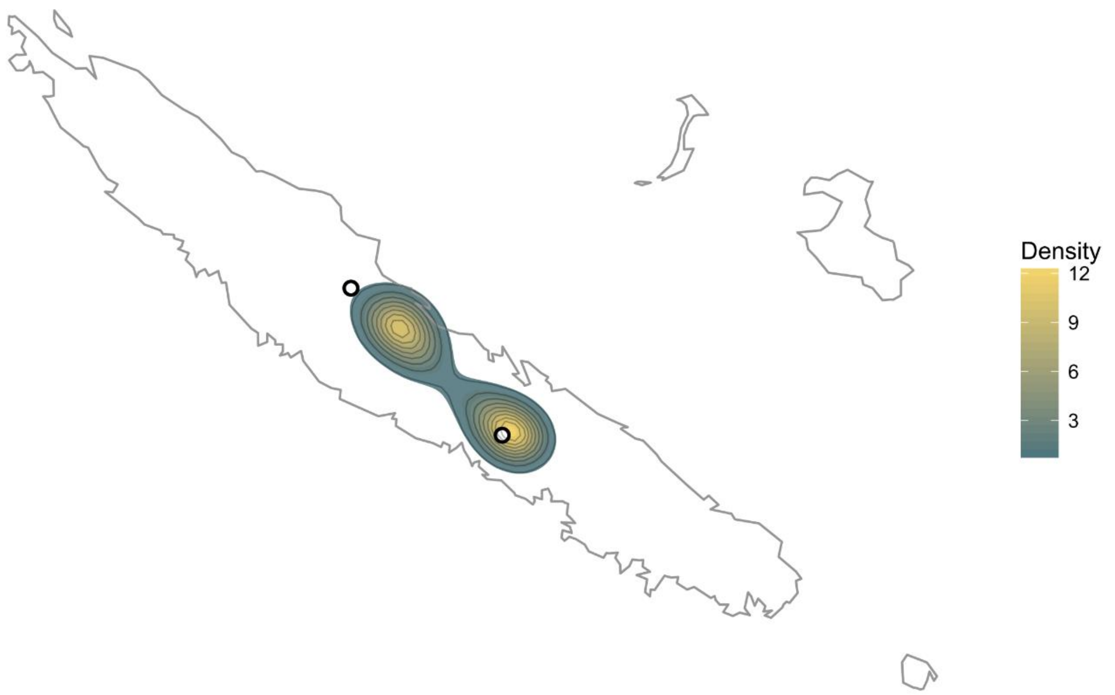

Figure – Posterior density of the expansion origins. White dots represent the true locations of expansion origins.

Table – Great-circle error distances (GCED, in kilometres) between true and estimated locations of South and North expansion origins, estimated with 200 cross-validations.

	GCED South (km)	GCED North (km)
**Mean**	8.24	27.33
**Median**	6.19	26.20
**Standard Dev**	6.73	9.76

Conclusion. Homogenising the carrying capacities does not change significantly the inference of the locations of expansion origins (we may detect a slight bias for the North origin), but the geographical separation of the two origins is less marked. We expect therefore the inference of origin coordinates to be robust to the prior properties (zonal vs. homogeneous pattern) of the carrying capacity map.

1.3.6.3 Number of expansion origins: single origin

Our spatial model implements two independent expansion origins. How would the inference behave with this model, when we had a **single** expansion origin instead?

Method. Based on the posterior adjusted model, we generated 200 PODs assuming a single expansion origin, located in the South (white dot in the figure below). We performed ABC cross-validations (same procedure as for the aforementioned LOOCV), based on the model assuming 2 expansion origins.

Results.

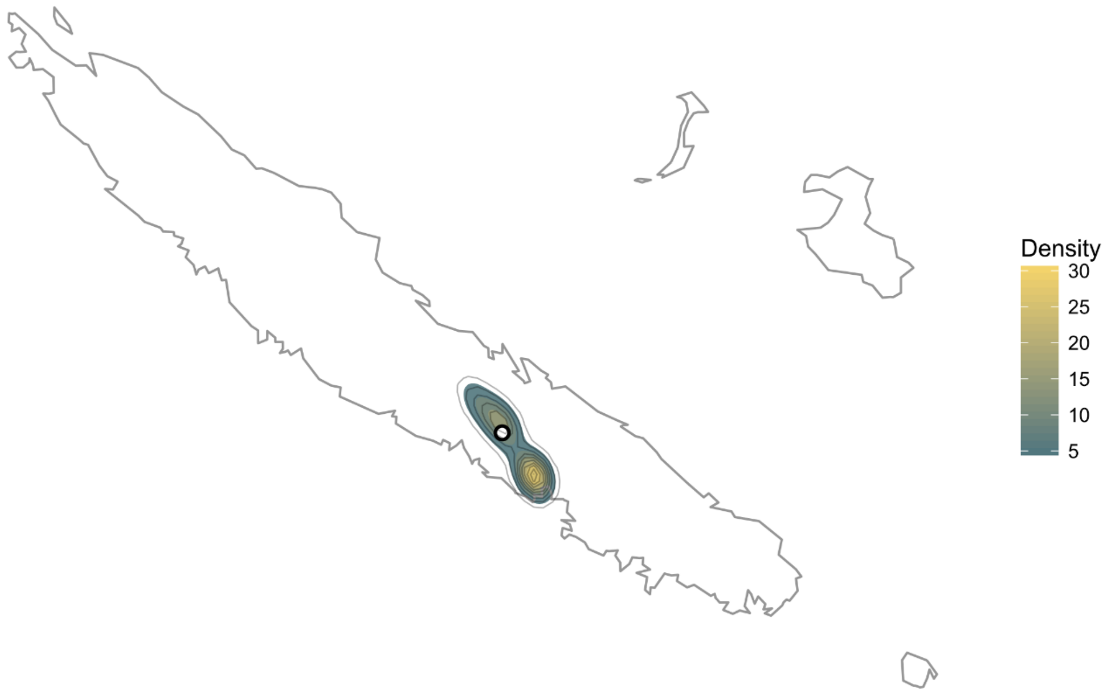

Figure - Posterior density of the expansion origins. The white dot represents the true locations of the **single** expansion origin.

Conclusion. In the case where we simulate one single expansion origin, the model is still able to recover this origin with a narrow posterior spatial extent. Implementing two origins in the model does not appear to bias the inference if there were actually a single expansion origin.

1.3.6.4 Number of expansion origins: three origins

How would the inference behave with the 2-origin model, when we had an actual **three**-expansion origins?

Method. Based on the posterior adjusted model, we generated 200 PODs assuming three expansion origins: two correspond to the posterior coordinate estimates and an additional

origin was located at Ponandou (white dots in the figure below). We performed ABC cross-validations (same procedure as for the aforementioned LOOCV), based on the model assuming 2 expansion origins.

Results.

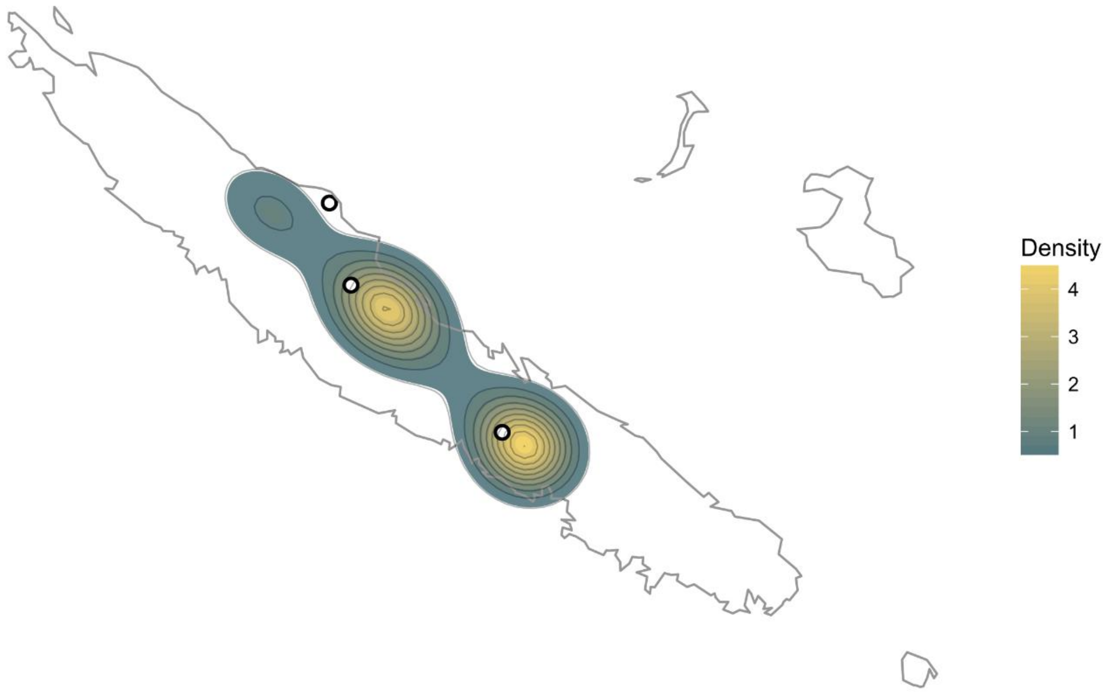

Figure – Posterior density of the expansion origins. White dots represent the true locations of the **three** expansion origins.

Conclusion. In the case where we simulate three expansion origins, the two-origin model seems to expand the northern inferred density. The northern distribution almost captures the three said origins. Hence, implementing only two origins widens the confidence interval of the inference in the model but does not seem to significantly bias the estimated distribution of expansion origins.

1.4 Species Distribution Modelling (SDM)

To compare our genetic model-based predictions of past distributions to more classical correlative species distribution modelling, we modelled habitat suitabilities for *Amborella trichopoda* in New Caledonia under different ages of the past—mid-Holocene (~6,000 BP) and the Last Glacial Maximum (LGM, ~18,000 BP)—by projecting the current distribution model of *Amborella trichopoda* as determined by Poncet et al. [14] under paleoclimate conditions. Paleo-distribution projection was performed with the MaxEnt software package [15].

1.4.1 Discretization of paleo-occurrence probabilities

Continuous logistic probabilities of paleo-occurrence were discretized into three categories: *unlikely presence* for probabilities below the maximum training sensitivity plus specificity cut-off (0.293), *likely presence* for probabilities above the equal training sensitivity and specificity cut-off (0.379) and *probable presence* for probabilities in-between. The cut-off values are derived from the MaxEnt receiver operating characteristic curve computed by Poncet et al. [14].

2 Supplementary references

1. Frichot E, Mathieu F, Trouillon T, Bouchard G, François O (2014) Fast and Efficient Estimation of Individual Ancestry Coefficients. Genetics 196: 973-983.

2. Alexander DH, Novembre J, Lange K (2009) Fast model-based estimation of ancestry in unrelated individuals. Genome Research 19: 1655-1664.

3. Weir BS, Cockerham CC (1984) Estimating F-Statistics for the Analysis of Population Structure. Evolution 38: 1358-1370.

4. Danecek P, Auton A, Abecasis G, Albers CA, Banks E, et al. (2011) The variant call format and VCFtools. Bioinformatics 27: 2156-2158.

5. Mountain JL, Cavalli-Sforza LL (1997) Multilocus genotypes, a tree of individuals, and human evolutionary history. Am J Hum Genet 61: 705-718.

6. Excoffier L, Dupanloup I, Huerta-Sánchez E, Sousa VC, Foll M (2013) Robust demographic inference from genomic and SNP data. PLoS Genet 9: e1003905.

7. Mazet O, Rodriguez W, Grusea S, Boitard S, Chikhi L (2016) On the importance of being structured: instantaneous coalescence rates and human evolution[mdash]lessons for ancestral population size inference[quest]. Heredity 116: 362-371.

8. Maisano Delser P, Corrigan S, Hale M, Li C, Veuille M, et al. (2016) Population genomics of C. melanopterus using target gene capture data: demographic inferences and conservation perspectives. Scientific Reports 6: 33753.

9. Ray N, Currat M, Foll M, Excoffier L (2010) SPLATCHE2: a spatially explicit simulation framework for complex demography, genetic admixture and recombination. Bioinformatics 26: 2993-2994.

10. Kimmel M, Chakraborty R (1996) Measures of variation at DNA repeat loci under a general stepwise mutation model. Theoretical Population Biology 50: 345-367.

11. Csilléry K, Blum MG, Gaggiotti OE, Francois O (2010) Approximate Bayesian Computation (ABC) in practice. Trends in Ecology and Evolution 25: 410-418.

12. Blum MGB, Nunes MA, Prangle D, Sisson SA (2013) A Comparative Review of Dimension Reduction Methods in Approximate Bayesian Computation. 189-208.

13. Csilléry K, François O, Blum MGB (2012) abc: an R package for approximate Bayesian computation (ABC). Methods in Ecology and Evolution 3: 475-479.

14. Poncet V, Munoz F, Munzinger J, Pillon Y, Gomez C, et al. (2013) Phylogeography and niche modelling of the relict plant Amborella trichopoda (Amborellaceae) reveal multiple Pleistocene refugia in New Caledonia. Molecular Ecology 22: 6163-6178.

15. Phillips SJ, Anderson RP, Schapire RE (2006) Maximum entropy modeling of species geographic distributions. Ecological Modelling 190: 231-259.
