# Supplementary material for: Two disjunct Pleistocene populations and anisotropic postglacial expansion shaped the current genetic structure of the relict plant Amborella trichopoda
Source: PLoS One. 2017 Aug 18;12(8):e0183412. doi: 10.1371/journal.pone.0183412 (PMC5562301; doi:10.1371/journal.pone.0183412)
Supplement: S1 Table — (PDF) [file pone.0183412.s003.pdf]

S1 Table. Genetic sampling design.

Population	Latitude[c]	Longitude[c]	SAMPLE SIZES		
			Raw SSR dataset[a]	Filtered SSR dataset[b]	Whole-genome resequencing dataset[a]
Amieu	7610019.653	583889.5808	16	32	1
Aoupinié	7660520.957	533180.7683	27	51	1
Ba	7654287.134	556460.0972	11	17	1
Boregaou	7630222.625	553742.8979	13	26	1
Dogny	7608714.055	589929.3372	31	60	1
Mé Foméchawa	7617318.384	575332.0226			1
Mé Ori	7618405.476	570170.3067	12	22	1
Nakada[d]	7607044.021	609343.6338	37	74	
Ponandou	7698038.907	522890.1535	14	25	1
Pwicaté	7683197.481	491324.56	26	52	1
Tchamba	7676804.964	524928.0609	9	18	1
Tonine	7698444.956	510146.0048	26	42	1
Santa Cruz	Grown in the Santa Cruz Conservatorium (USA), likely sampled from a wild population near Mé Foméchawa				1
**TOTAL**			**222**	**419**	**12**

[a] Number of diploid individuals.

[b] Number of haploid individuals.

[c] UTM 58S.

[d] Nakada population, representing an original and distinct genetic group according to Poncet et al. (2013), was not considered in the analyses based on the whole-genome resequencing dataset because it would have been excessively under-sampled (single individual).
