# Supplementary material for: Two disjunct Pleistocene populations and anisotropic postglacial expansion shaped the current genetic structure of the relict plant Amborella trichopoda
Source: PLoS One. 2017 Aug 18;12(8):e0183412. doi: 10.1371/journal.pone.0183412 (PMC5562301; doi:10.1371/journal.pone.0183412)
Supplement: S2 Table — (PDF) [file pone.0183412.s004.pdf]

S2 Table. Matrix of pairwise allele sharing distances.

	Amieu	Aoupinie	Ba	Boregaou	Dogny	MeFo	MeOri	Ponandou	Pwicate	Tchamba	Tonine
Amieu											
Aoupinie	0.340										
Ba	0.311	0.311									
Boregaou	0.245	0.350	0.245								
Dogny	0.211	0.366	0.318	0.235							
MeFo	0.282	0.407	0.349	0.284	0.293						
MeOri	0.239	0.386	0.315	0.224	0.239	0.206					
Ponandou	0.444	0.295	0.433	0.456	0.463	0.513	0.495				
Pwicate	0.448	0.291	0.431	0.462	0.462	0.509	0.494	0.223			
Tchamba	0.397	0.257	0.379	0.406	0.420	0.462	0.445	0.228	0.235		
Tonine	0.462	0.303	0.428	0.473	0.479	0.514	0.504	0.186	0.227	0.261	
SantaCruz	0.300	0.452	0.370	0.290	0.298	0.176	0.168	0.567	0.570	0.515	0.571
