# Supplementary material for: Two disjunct Pleistocene populations and anisotropic postglacial expansion shaped the current genetic structure of the relict plant Amborella trichopoda
Source: PLoS One. 2017 Aug 18;12(8):e0183412. doi: 10.1371/journal.pone.0183412 (PMC5562301; doi:10.1371/journal.pone.0183412)
Supplement: S3 Table — (PDF) [file pone.0183412.s005.pdf]

S3 Table. Typology of the non-spatial demo-genomic models.

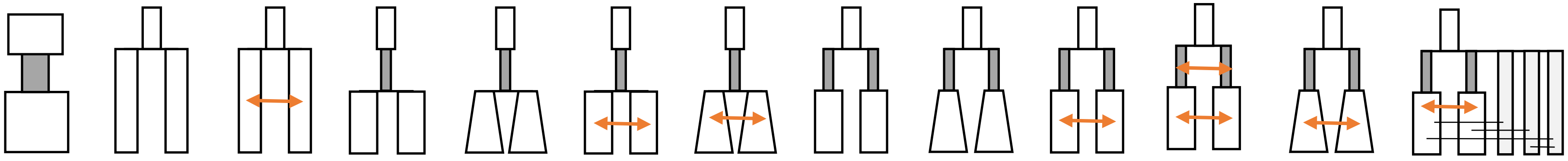

	Po	Do	DoM	1	1G	1M	1MG	2	2G	2M	2MM	2MG	3I
Number of expansion origins	1	0	0	1	1	1	1	2	2	2	2	2	2
Recent gene flow	No	No	Yes	No	No	Yes	Yes	No	No	Yes	Yes	Yes	Yes
Gene flow between ancestral populations	No	No	No	No	No	No	No	No	No	No	Yes	No	No
Exponential expansion	No	No	No	No	Yes	No	Yes	No	Yes	No	No	Yes	No
