# Supplementary material for: Two disjunct Pleistocene populations and anisotropic postglacial expansion shaped the current genetic structure of the relict plant Amborella trichopoda
Source: PLoS One. 2017 Aug 18;12(8):e0183412. doi: 10.1371/journal.pone.0183412 (PMC5562301; doi:10.1371/journal.pone.0183412)
Supplement: S4 Table — Maximum composite likelihoods, AICs and evidence ratios for the 13 tested demo-genomic models. (PDF) [file pone.0183412.s006.pdf]

S4 Table. Non spatial demo-genomic model comparison. Maximum composite likelihoods, AICs and evidence ratios for the 13 tested demo-genomic models.

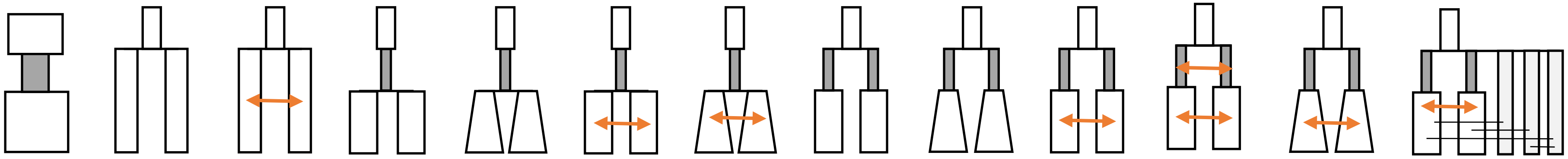

	Po	Do	DoM	1	1G	1M	1MG	2	2G	2M	2MM	2MG	3I
**ln(L)**[a]	-1269285	-1175486	-1170663	-1175141	-1174252	-1170585	-1169175	-1174292	-1174221	-1168256	-1168293	-1168546	-1168180
**AIC**	2538583	2350983	2341339	2350297	2348522	2341188	2338372	2348601	2348462	2336532	2336610	2337115	2336384
**w_1**[b]	0	0	0	0	0	0	0	0	0	1	1.15E-17	2.53E-127	
**w_2**[b]	0	0	0	0	0	0	0	0	0	7.28E-33	8.41E-50	1.84E-159	1

[a] Logarithm of the maximum composite likelihood of the fittest run for each model.

[b] Evidence ratio (w_1) including the **3I** model; (w_2) or not. The formula to calculate the evidence ratio is:

$$w_i = e^{-\Delta_i/2} \div \sum_{m=1}^{M} e^{-\Delta_m/2}$$

with m the model index and $\Delta_i = AIC_i - AIC_{min}$, where AIC_{min} is the overall minimum AIC across all independent runs and candidate models.
