# Supplementary material for: Two disjunct Pleistocene populations and anisotropic postglacial expansion shaped the current genetic structure of the relict plant Amborella trichopoda
Source: PLoS One. 2017 Aug 18;12(8):e0183412. doi: 10.1371/journal.pone.0183412 (PMC5562301; doi:10.1371/journal.pone.0183412)
Supplement: S5 Table — (PDF) [file pone.0183412.s007.pdf]

**S5 Table. Parameter estimation for the most likely demo-genomic model (2M).**

| Parameter | Point estimate | Confidence Interval (95%) | Definition |
|---|---|---|---|
| **NeNorth** | 10,136 | 4,051 – 38,040 | Current haploid effective size of the North population |
| **NeSouth** | 9,834 | 3,684 – 38,560 | Current haploid effective size of the South population |
| **TBottle** | 3,197 | 1,625 – 9,760 | Age (generations BP) of the expansion onset |
| **NeBottle_North** | 10,101 | 2,173 – 29,986 | Haploid effective size of the North ancestral population |
| **NeBottle_South** | 1,548 | 52 – 9,676 | Haploid effective size of the South ancestral population |
| **TSplit** | 5,844 | 3,425 – 21,943 | Age (generations BP) of the divergence between the North and South ancestral populations |
| **NeAncestral** | 10,464 | 146 – 40,450 | Haploid effective size of the earliest population |
| **M[N➔S]** | 1.06E-4 | 1.17E-5 – 1 .43E-4 | Per-generation migration rate from North to South present populations |
| **M[S➔N]** | 3.48E-5 | 2.83E-5 – 2 .48E-4 | Per-generation migration rate from South to North present populations |
| **MutationRate** | 4.15E-8 | 9.14E-9 – 8. 35E-8 | Number of neutral substitutions per site per generation |
