# Supplementary material for: Two disjunct Pleistocene populations and anisotropic postglacial expansion shaped the current genetic structure of the relict plant Amborella trichopoda
Source: PLoS One. 2017 Aug 18;12(8):e0183412. doi: 10.1371/journal.pone.0183412 (PMC5562301; doi:10.1371/journal.pone.0183412)
Supplement: S6 Table — Genetic summary statistics derived from the observed probabilistic SFS vs. their prediction under the adjusted 2M scenario. (PDF) [file pone.0183412.s008.pdf]

S6 Table. Predictive check of the adjusted 2M demo-genomic model.

Genetic summary statistics derived from the observed probabilistic SFS vs. their prediction under the adjusted 2M scenario.

		Observed	Predicted (mean)	Predicted (CI95% low)[g]	Predicted (IC95% up)
**Singlet**[a]	North	6.48E-04	6.94E-04	6.83E-04	7.06E-04
	South	7.54E-04	7.39E-04	7.30E-04	7.48E-04
**Private**[b]	North	1.30E-03	1.32E-03	1.31E-03	1.34E-03
	South	1.52E-03	1.53E-03	1.51E-03	1.54E-03
**Shared**[c]		1.18E-03	1.14E-03	1.13E-03	1.15E-03
**S**[d]	North	2.47E-03	2.46E-03	2.45E-03	2.48E-03
	South	2.69E-03	2.66E-03	2.64E-03	2.68E-03
**H_S**[e]	North	0.2272304	0.2266182	0.225501	0.2277367
	South	0.1882863	0.1892305	0.1883246	0.1901639
**F_{ST}**[f]		0.1599501	0.1598357	0.1577013	0.1619462

[a] Proportion of singleton SNPs (over all sites).

[b] Proportion of private SNPs (over all sites).

[c] Proportion of shared SNPs (over all sites).

[d] Proportion of SNPs over all sites.

[e] Expected heterozygosity.

[f] Wright's F_{ST}.

[g] Lower bound of the confidence interval at 95%.
