# Supplementary material for: Two disjunct Pleistocene populations and anisotropic postglacial expansion shaped the current genetic structure of the relict plant Amborella trichopoda
Source: PLoS One. 2017 Aug 18;12(8):e0183412. doi: 10.1371/journal.pone.0183412 (PMC5562301; doi:10.1371/journal.pone.0183412)
Supplement: S7 Table — (PDF) [file pone.0183412.s009.pdf]

S7 Table. Mean-standardized root-mean-square error (SRMSE) of parameter estimation for the spatial coalescent model.

	Mean-standardized root mean square error (SRMSE)
CARCAP1 (North)	72.52%
CARCAP2 (Central)	64.44%
CARCAP3 (South)	9.91%
COORD_SOURCE_1[a]	0.28%
COORD_SOURCE_1_LONG[a]	3.60%
COORD_SOURCE_2[b]	0.28%
COORD_SOURCE_2_LONG[b]	4.76%
GAMMA	11.38%
MID.ALT	57.69%
MUTRATE	5.04%
SOURCE1.Ne[a]	42.97%
SOURCE2.Ne[b]	50.31%
GROWTHRATE	78.51%
MIGRATIONRATE	23.45%
PLAIN.F.COEF.VAL	63.68%
SOMMIT.F.COEF.VAL	82.67%

[a] South population
[b] North population
