# Supplementary material for: Two disjunct Pleistocene populations and anisotropic postglacial expansion shaped the current genetic structure of the relict plant Amborella trichopoda
Source: PLoS One. 2017 Aug 18;12(8):e0183412. doi: 10.1371/journal.pone.0183412 (PMC5562301; doi:10.1371/journal.pone.0183412)
Supplement: S8 Table — (PDF) [file pone.0183412.s010.pdf]

S8 Table. Mean posterior estimates for key parameters of the spatially explicit demo-genetic model.

Forward expansion parameters	Mean posterior estimate	95% credible interval
Carrying capacity of the Northern zone, K_N[a]	558	62–1,334
Carrying capacity of the Central zone, K_C[a]	826	134–1,417
Carrying capacity of the Southern zone, K_S[a]	24	4–177
Intrinsic growth rate, *r*	0.468	0.452–9.60
Intrinsic stepwise migration rate, *m*	0.0262	0.0039–0.2150
Elevational threshold (meters) [b]	607	36–1,360

[a] Carrying capacities are given as the number of diploid individuals per hectare.

[b] The elevational threshold is a parameter separating two different trends of the gene flow relative to the topography.
