# Supplementary figures and images for: Two disjunct Pleistocene populations and anisotropic postglacial expansion shaped the current genetic structure of the relict plant Amborella trichopoda
Source: PLoS One. 2017 Aug 18;12(8):e0183412. doi: 10.1371/journal.pone.0183412 (PMC5562301; doi:10.1371/journal.pone.0183412)

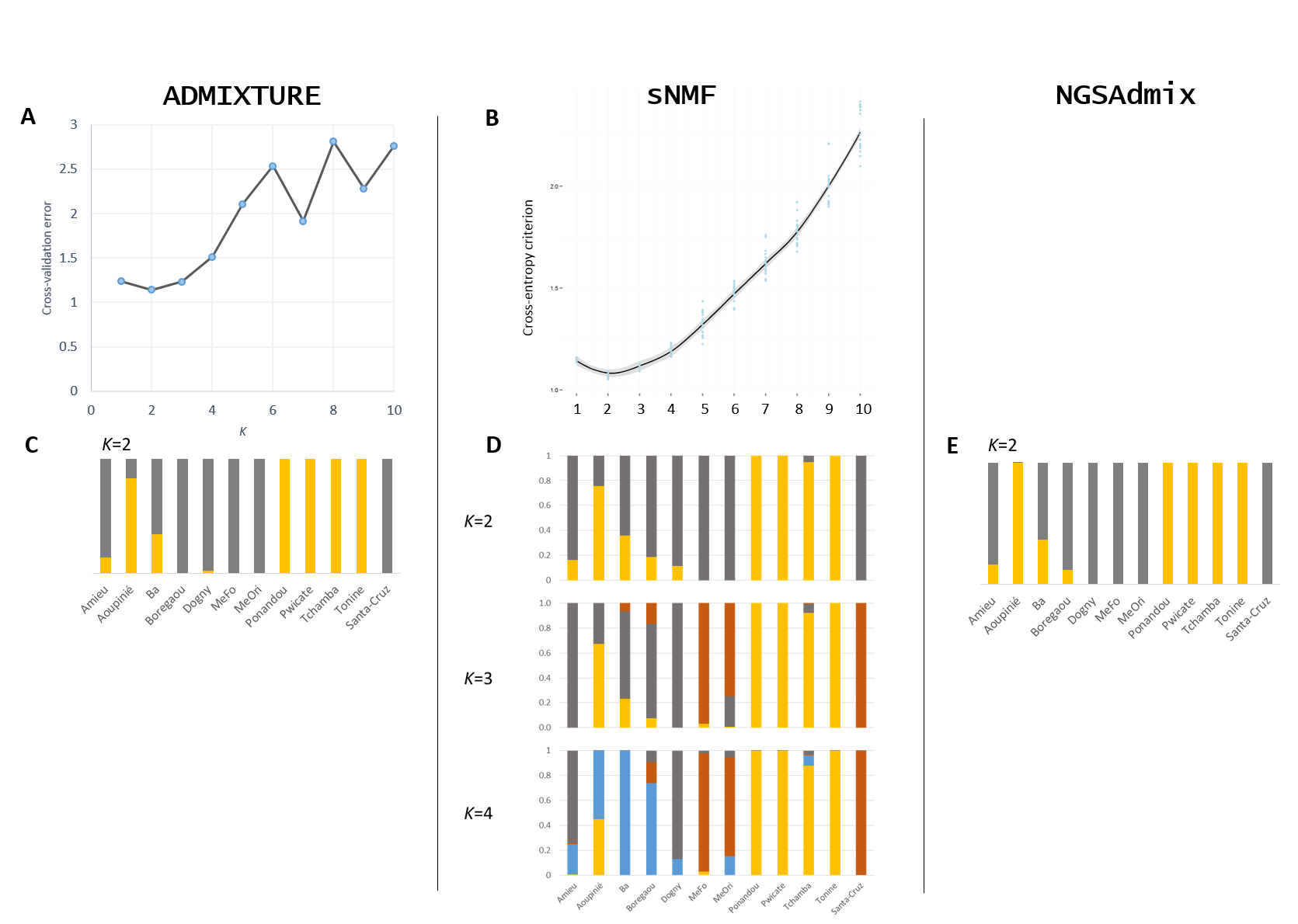

Supplement: S1 Fig — (A; C) Inference using the ADMIXTURE software; (B; D) using the sNMF software; (E) using the full-genotype likelihood software NGSAdmix. (TIF) [file pone.0183412.s011.tif]

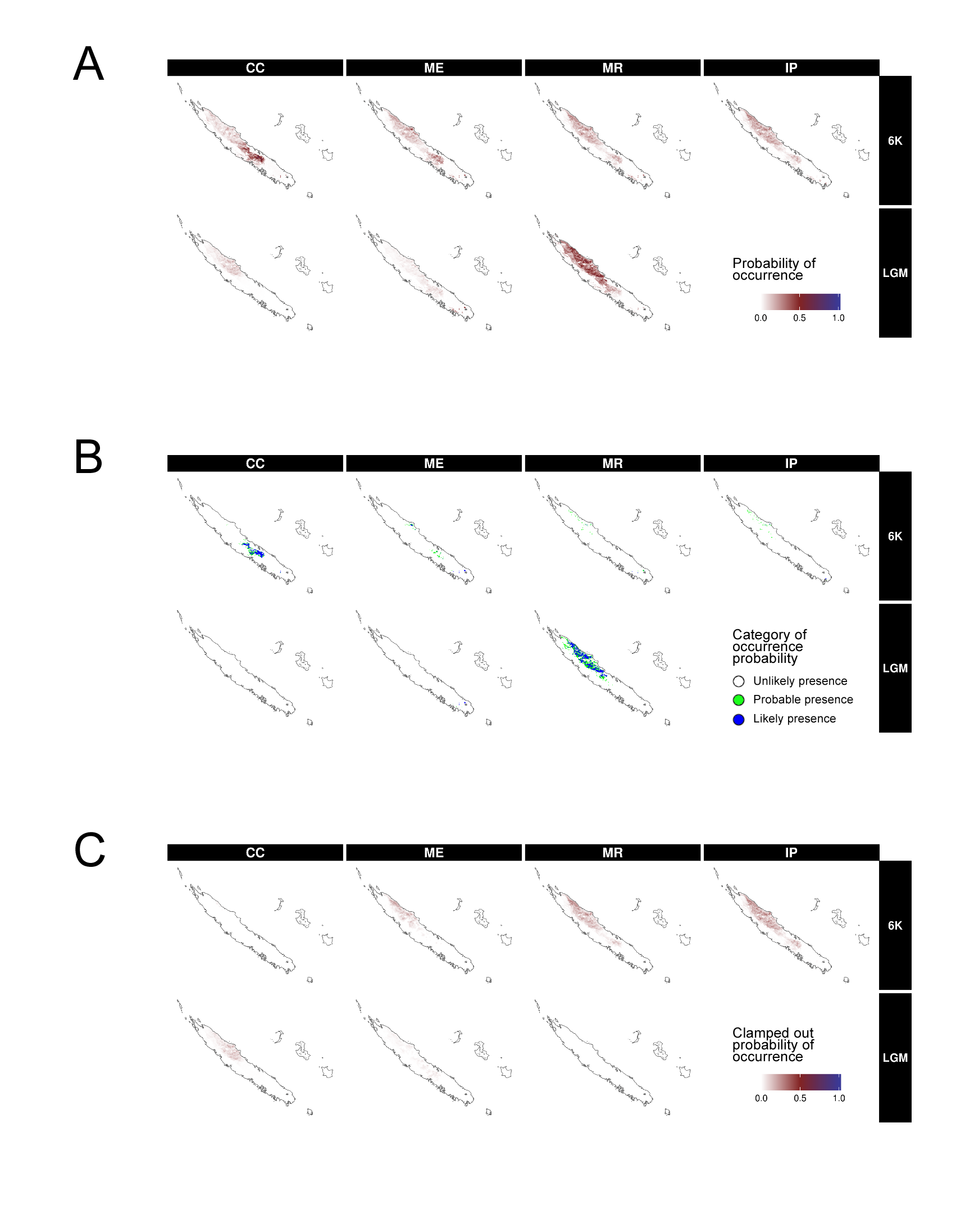

Supplement: S4 Fig — Maps of paleo-occurrence probabilities during the mid-Holocene (6,000 years BP) and during the Last Glacial Maximum (21,000 BP), based on the simulations of four different global circulation models: CCSM4 (CC), MPI-ESM-P (ME), MIROC-ESM (MR) and IPSL-CM5A-LR (IP). (A) Continuous logistic probabilities of paleo-occurrence; (B) categories of paleo-occurrence probability, defined using two previously published ROC-based cut-off values; (C) same as (A), but following a clamping procedure which down-weighted the probabilities where paleo-climatic conditions fell out of the climatic range represented in the training set at present. (TIF) [file pone.0183412.s014.tif]
